# Supplementary material for: Current genomic deep learning models display decreased performance in cell type-specific accessible regions
Source: Genome Biol. 2024 Aug 1;25:202. doi: 10.1186/s13059-024-03335-2 (PMC11293111; doi:10.1186/s13059-024-03335-2)
Supplement: Supplementary file 1 — Additional file 1: Supplementary figures S1–S19. [file 13059_2024_3335_MOESM1_ESM.pdf]

Supplementary Figures

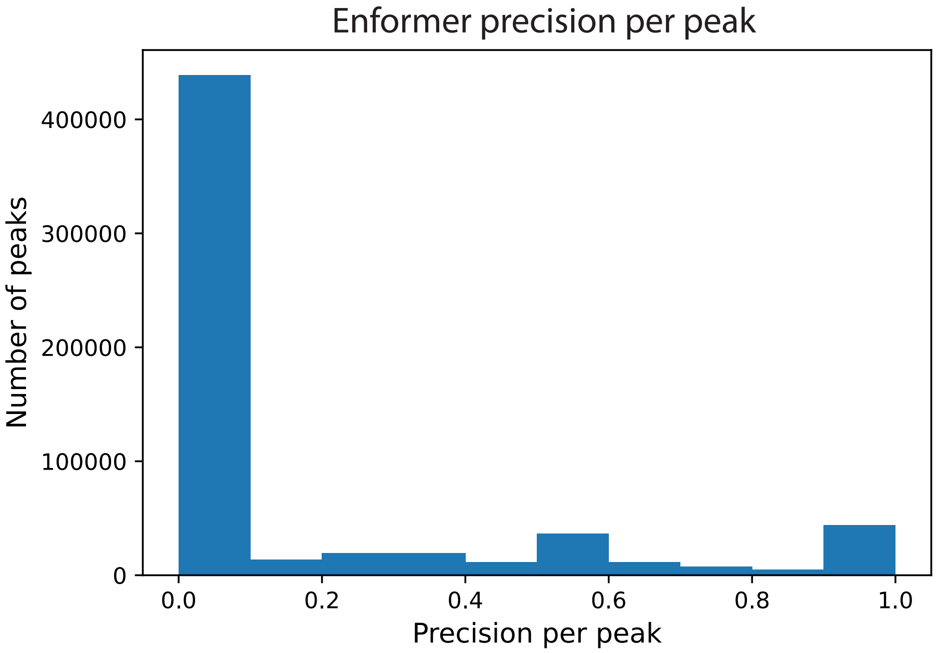

Fig. S1 Cell type precision of Enformer's chromatin accessibility predictions. For all sequences in Enformer's test set that are a peak in at least one experimental chromatin accessibility track, we compute the precision of Enformer's predictions across chromatin accessibility tracks. For each peak sequence, we first identify the true number (N) of experimental chromatin accessibility tracks with a peak. Then, based on the N chromatin accessibility tracks Enformer predicts to have the highest accessibility for that peak, we compute the precision of its prediction. To ensure an even comparison across tracks, we compare z-score normalized predictions for each track.

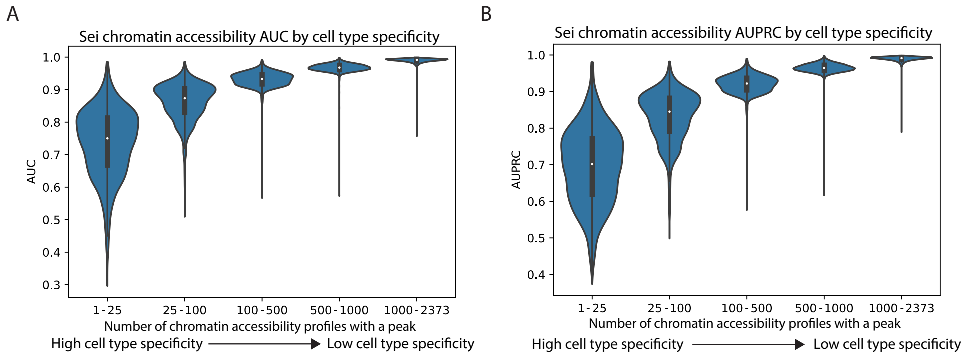

Fig. S2 Sei chromatin accessibility prediction performance stratified by cell type specificity. Sei's chromatin accessibility prediction A) AUROC and B) AUPRC measured for sequences in accessibility peak bins with varying degrees of cell type specificity. Sei's chromatin accessibility predictions are highly accurate in low cell type specificity peaks and less accurate in high cell type specificity peaks.

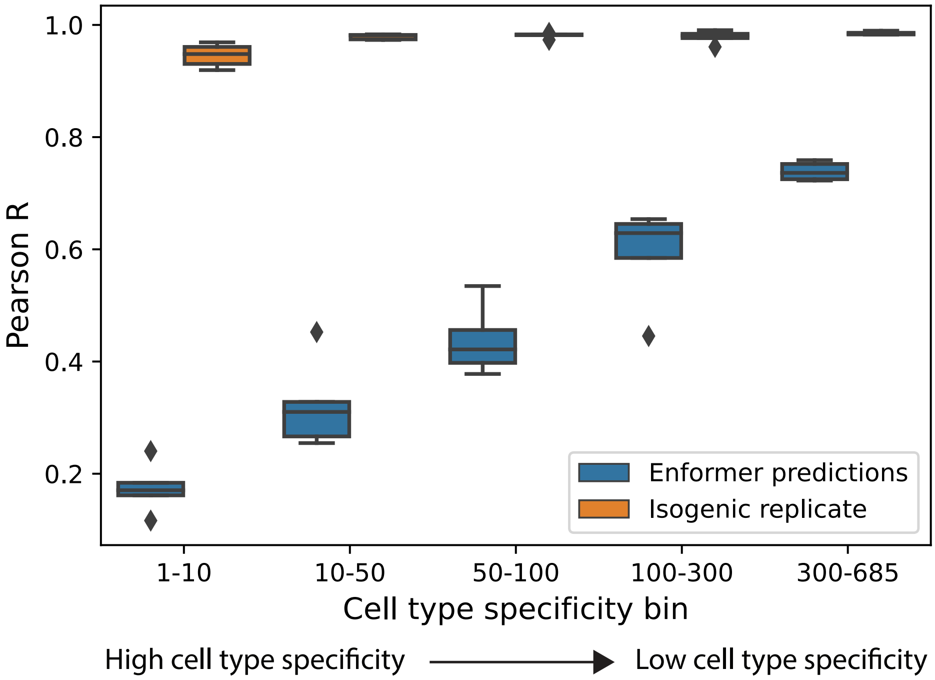

Fig. S3 Comparison of Enformer prediction performance versus isogenic replicate correlation for peaks with varying degrees of cell type specificity. For five representative Enformer DNase tracks with isogenic replicate data available on ENCODE, the peak height correlation between experimental replicates remains high even for high cell type specificity peaks. Thus experimental noise does not explain the dramatic drop in Enformer's predictive performance for cell type specific peaks.

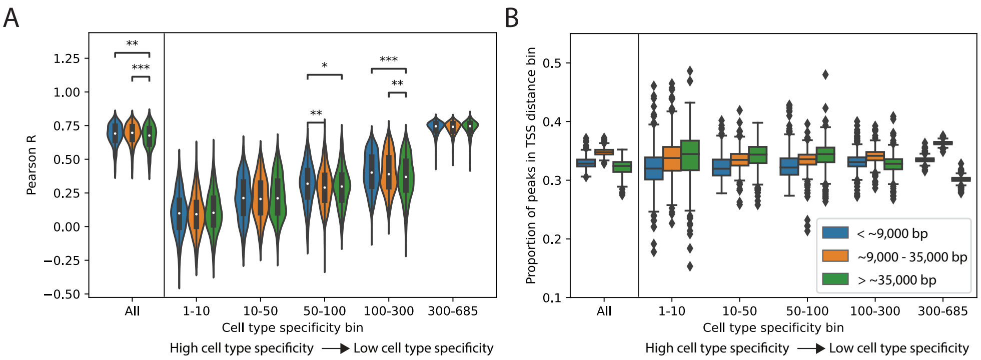

Fig. S4 Enformer chromatin accessibility prediction performance stratified by cell type specificity and TSS distance. A) Enformer prediction performance for peaks stratified by TSS distance only (left, "All") and for peaks stratified by both TSS distance and cell type specificity. B) The relative proportion of proximal versus distal sequences in each cell type specificity bin.

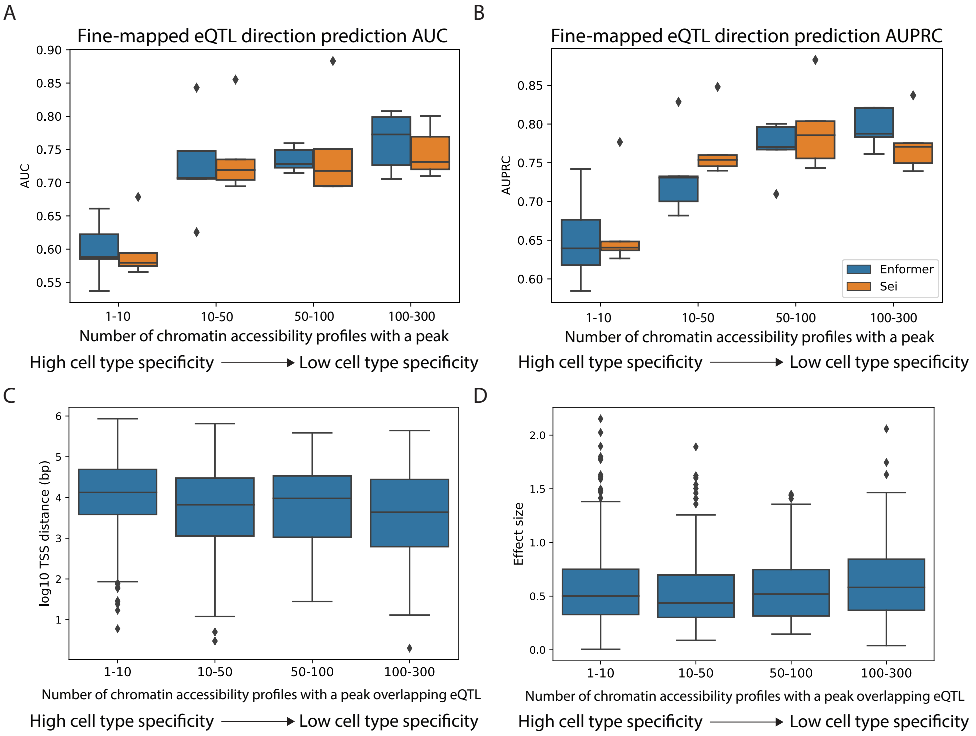

Fig. S5 Enformer and Sei eQTL direction prediction performance, TSS distance distribution, and effect size distribution stratified by cell type specificity. Enformer and Sei's eQTL direction prediction A) AUROC and B) AUPRC measured for high posterior inclusion probability eQTLs (PIP > 0.9) residing in accessibility peaks with varying degrees of cell type specificity. C) Distance to the nearest gene transcription start site (TSS) distributions and D) eQTL effect size distributions for high PIP eQTLs in each cell type specificity bin.

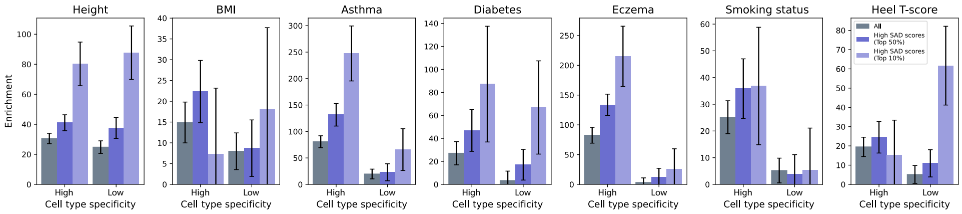

Fig. S6 Trait heritability enrichment of Enformer high SAD score variants is robust to choice of threshold. To verify that the results in Fig. 2D are robust to the choice of threshold, here we subset variants by taking only the top 10% highest Enformer SAD scores and assess enrichment of trait heritability in this subset.

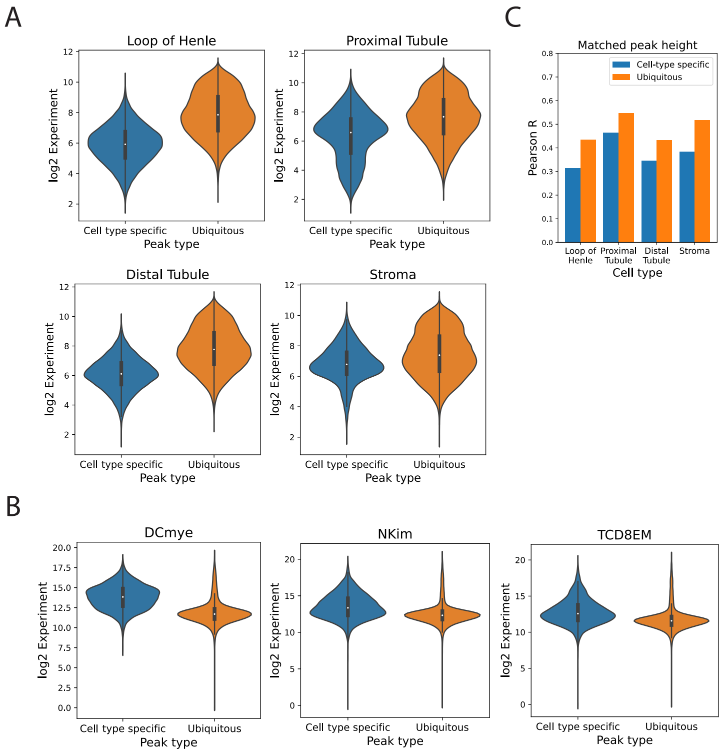

Fig. S7 Chromatin accessibility prediction performance in peak-height matched cell type specific and ubiquitous peaks. Peak height distributions for cell type specific and ubiquitous peaks in the A) Loeb et al. [27] and B) Calderon et al. [28] data. C) Predictive performance (reference accuracy) in cell type specific and ubiquitous peaks in the Loeb et al. [27] data after matching on peak height. Reference accuracy is measured as the Pearson correlation between experimentally measured versus predicted accessibility.

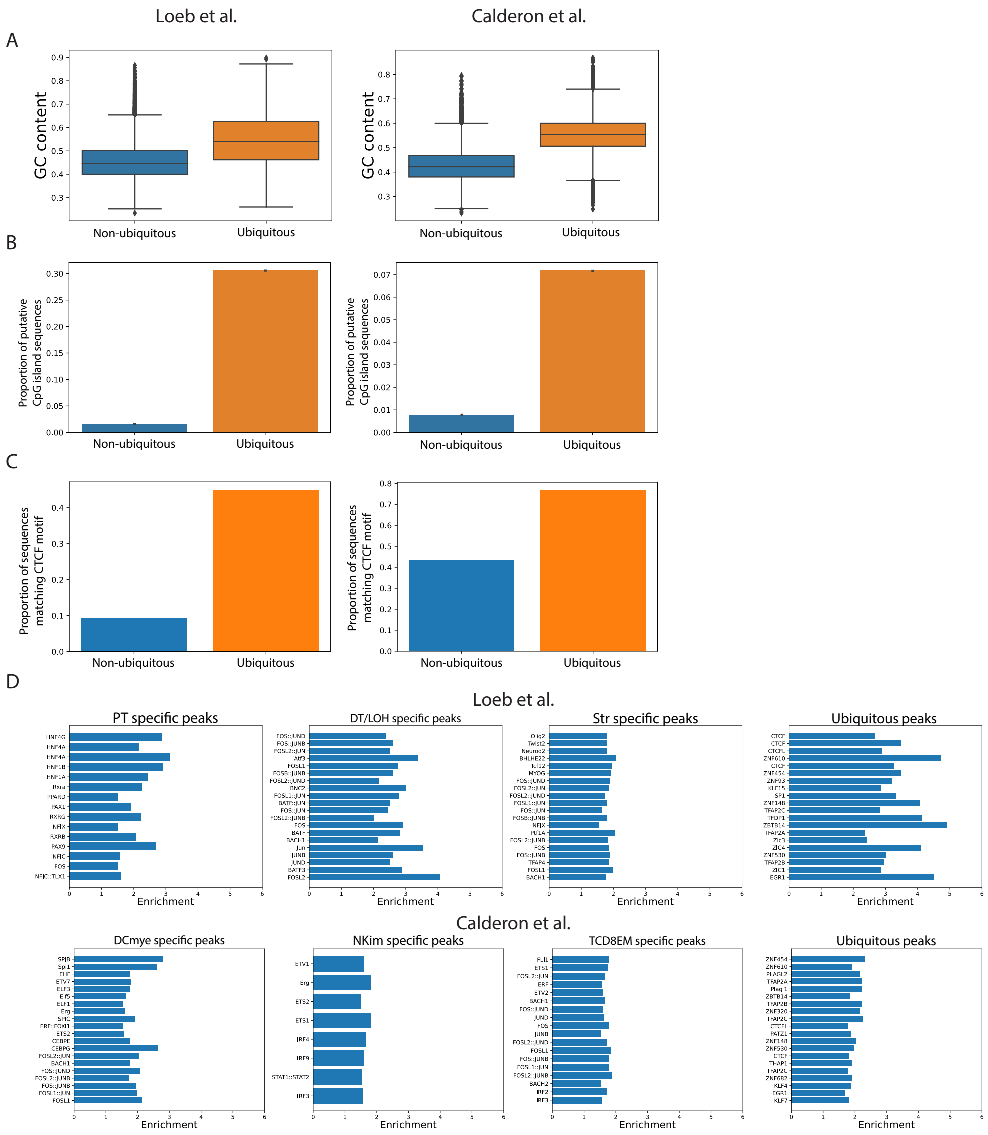

Fig. S8 Sequence features and top enriched motifs of ubiquitous and non-ubiquitous peaks in the Loeb et al. [27] and Calderon et al. [28] data. A) GC content fraction for sequences in ubiquitous and non-ubiquitous peaks. B) Proportion of sequences containing putative CpG islands, and C) proportion of sequences containing CTCF motifs in ubiquitous and non-ubiquitous peaks. D) Top motifs enriched in cell type specific and ubiquitous peaks relative to non-peaks. We show the enrichment of the top motifs that are present in at least 5% of the peaks in each peak set, have an enrichment p-value $\leq 10^{-6}$, and an enrichment greater than 1.5.

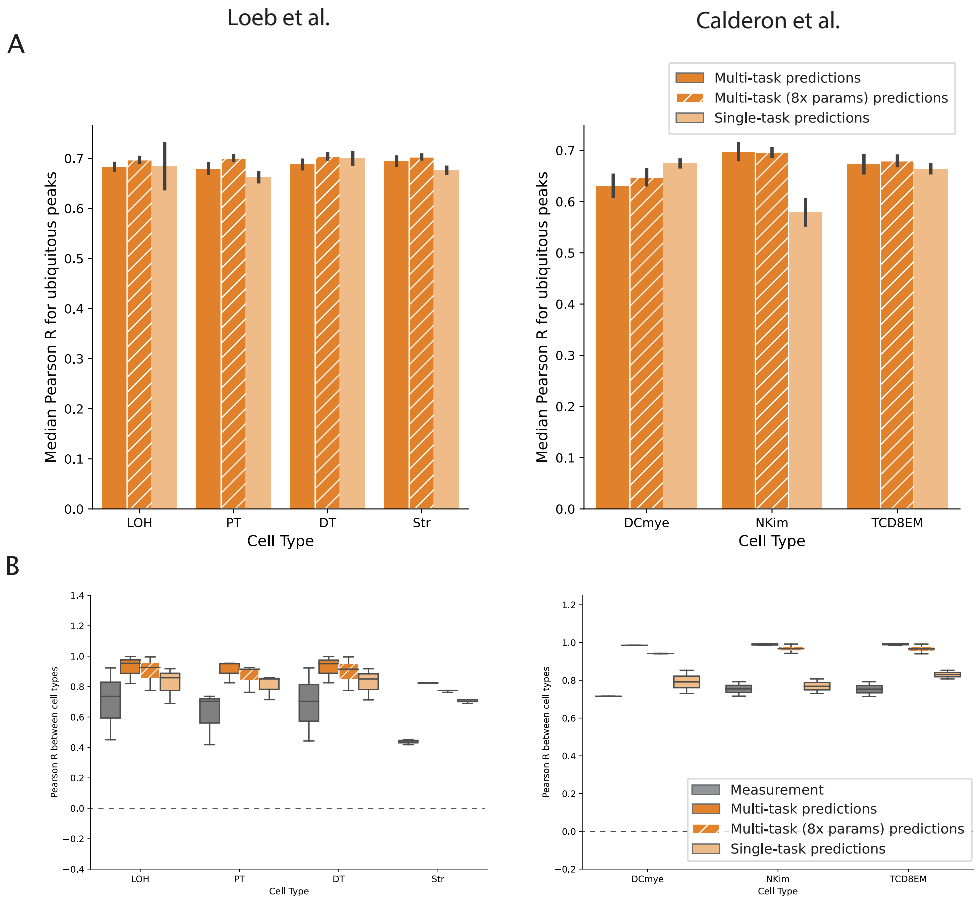

Fig. S9 Comparison of multi-task versus single-task model performance in ubiquitous peak regions. A) Reference accuracy of multi-task versus single-task models evaluated in ubiquitous peak regions. B) Pairwise correlations of peak height between cell types for experimental (gray) and model predicted accessibility (dark and light orange) in ubiquitous peaks. Model predicted accessibility is more correlated across cell types than experimentally measured accessibility, and this overcorrelation is slightly more pronounced in predictions from multi-task models than predictions from single-task models.

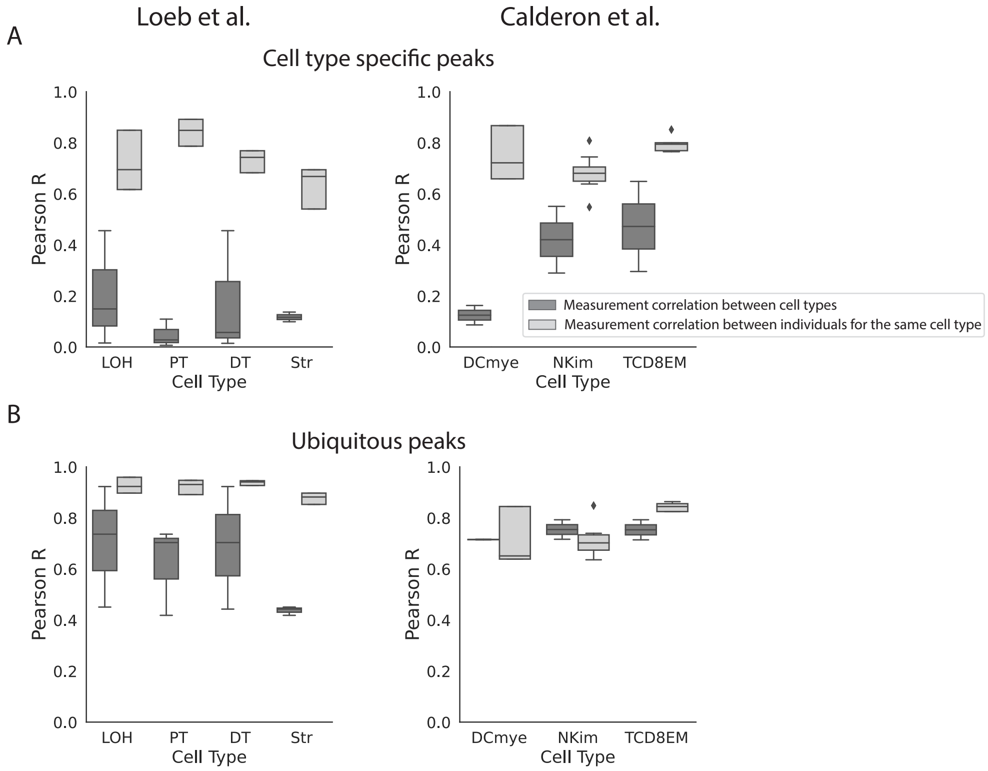

Fig. S10 Experimental measurement noise in cell type specific peaks does not explain the over-correlation in model predictions between cell types. A) Cell type specific peaks have low peak height correlation across cell types (dark gray), but high correlation across biological replicates (light gray). We note that because these biological replicates represent samples from different donors, they encompass both biological and experimental sources of variability and represent an upper bound on experimental noise. Thus experimental noise in cell type specific peaks does not explain the observed over-correlation of the model predictions across cell types (Fig. 4B). B) Peak heights of ubiquitous peaks are highly correlated both across cell types (dark gray) and across biological replicates (light gray).

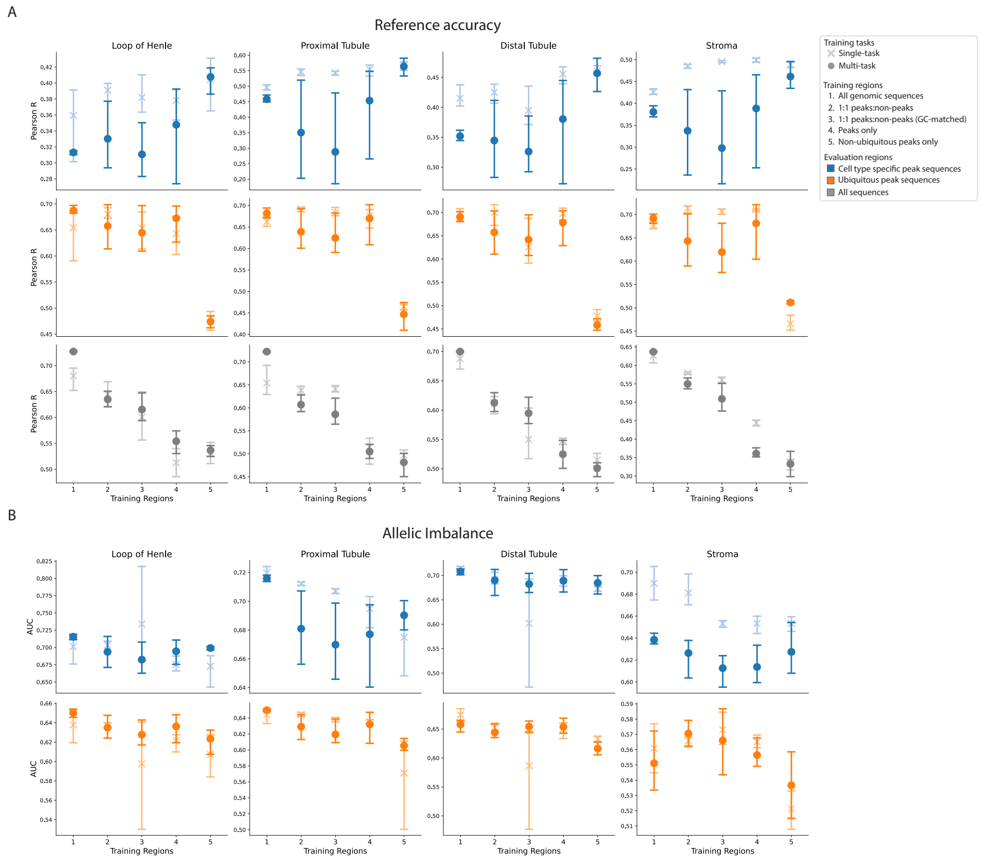

Fig. S11 Evaluating single-task versus multi-task learning and training set composition in the Loeb et al. [27] data. A) Reference accuracy (Pearson correlation between experimentally measured and predicted accessibility) and B) variant effect accuracy (AUROC for the model's ability to predict the direction of imbalance, or the higher accessibility allele, based on allelic imbalance measurements) in ubiquitous and cell type specific peak regions for the different modeling decisions that we evaluated.

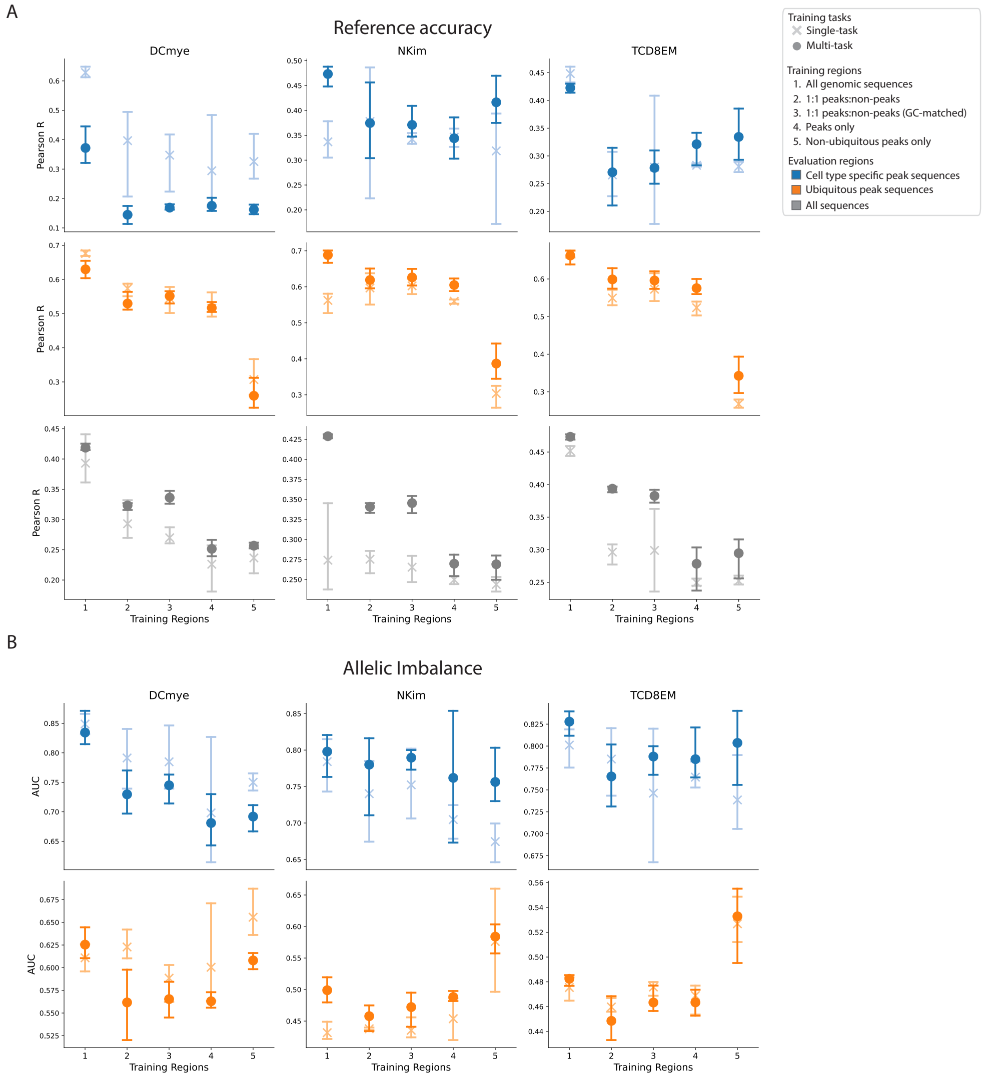

Fig. S12 Evaluating single-task versus multi-task learning and training set composition in the Calderon et al. [28] data A) Reference accuracy (Pearson correlation between experimentally measured and predicted accessibility) and B) variant effect accuracy (AUROC for the model's ability to predict the direction of imbalance, or the higher accessibility allele, based on allelic imbalance measurements) in ubiquitous and cell type specific peak regions for the different modeling decisions that we evaluated.

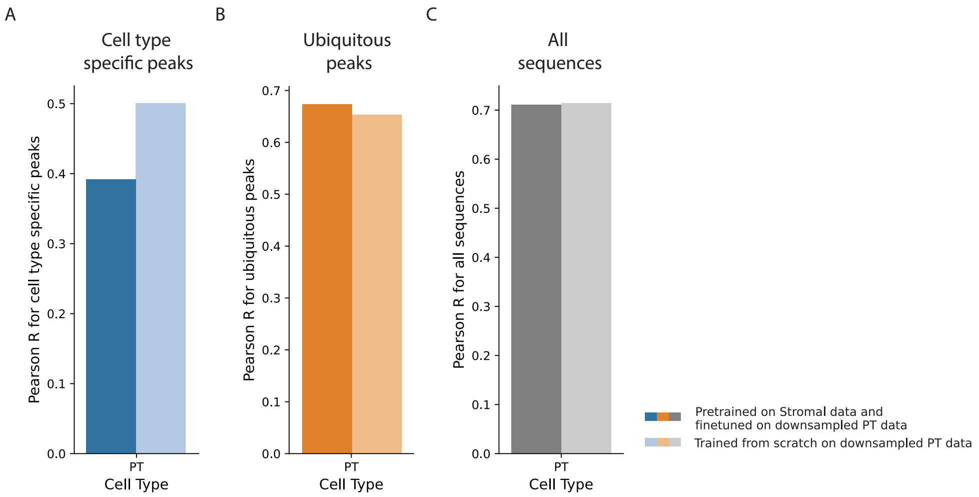

Fig. S13 Evaluation of a transfer learning strategy on cell type specific and ubiquitous peak prediction. Using the Loeb et al. [27] data, we evaluate a transfer learning strategy where a single-task model is first trained on a cell type with abundant high-quality data, and then fine-tuned on a cell type with lower quality data, reasoning that for cell types with lower quality data, this approach may retain beneficial aspects of both multi-task and single-task models. Specifically, we trained two additional model variants using a downsampled version of the Proximal Tubule data (downsampled to approximately 10% of the original number of collected cells): 1) a single-task model trained from scratch on the downsampled Proximal Tubule data (lighter colors), and 2) a single-task model pretrained on the Stromal cell data, and fine-tuned on the downsampled Proximal Tubule data (darker colors). We show the performance of both models on A) cell type specific peaks, B) ubiquitous peaks, and C) all genomic sequences.

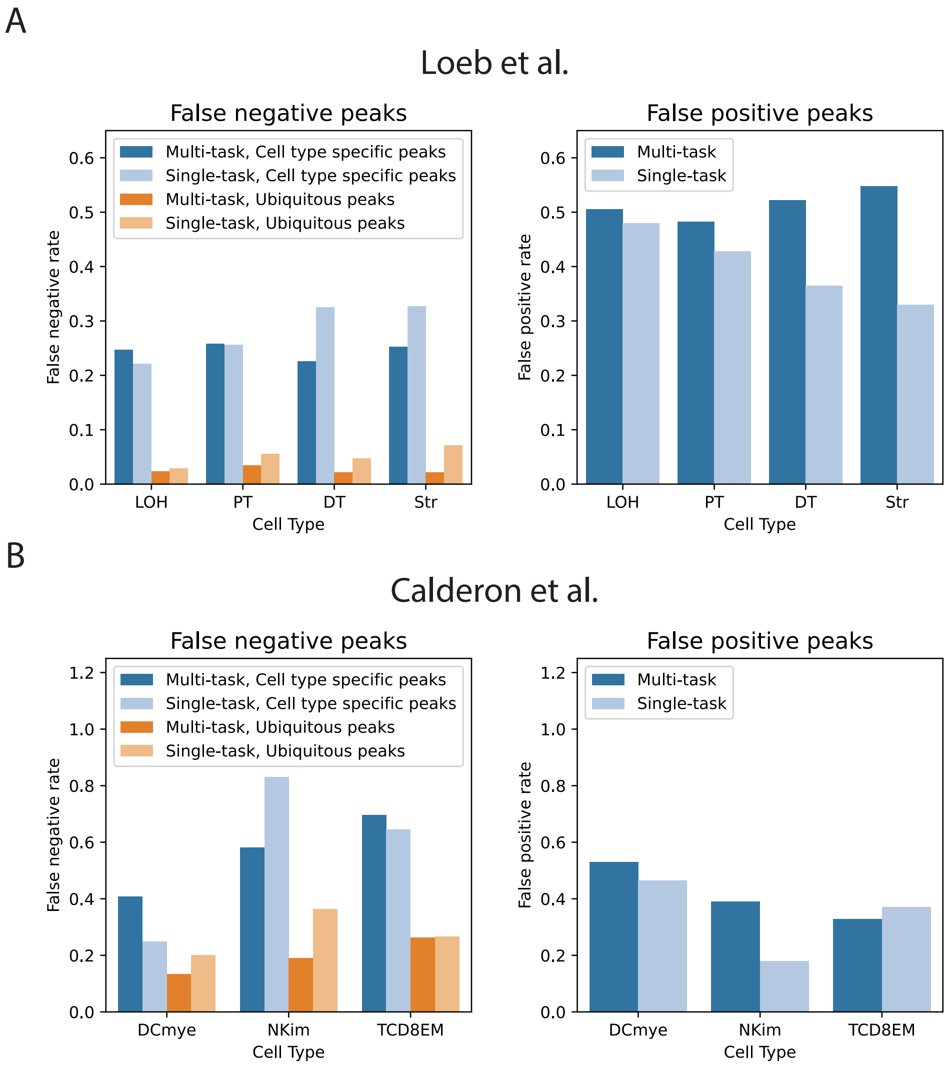

Fig. S14 Comparison of multi-task and single-task model false negative and false positive peak prediction rates. For both the A) Loeb et al. [27] and B) Calderon et al. [28] datasets, we compare the false negative and false positive rate of predictions from the baseline multi-task and single-task models. The baseline multi-task models tend to have higher false positive rates than single-task models, meaning that they predict that a peak is accessible in cell types in which the peak is not accessible. For both types of models, the false negative rate is higher for cell type specific peaks than ubiquitous peaks.

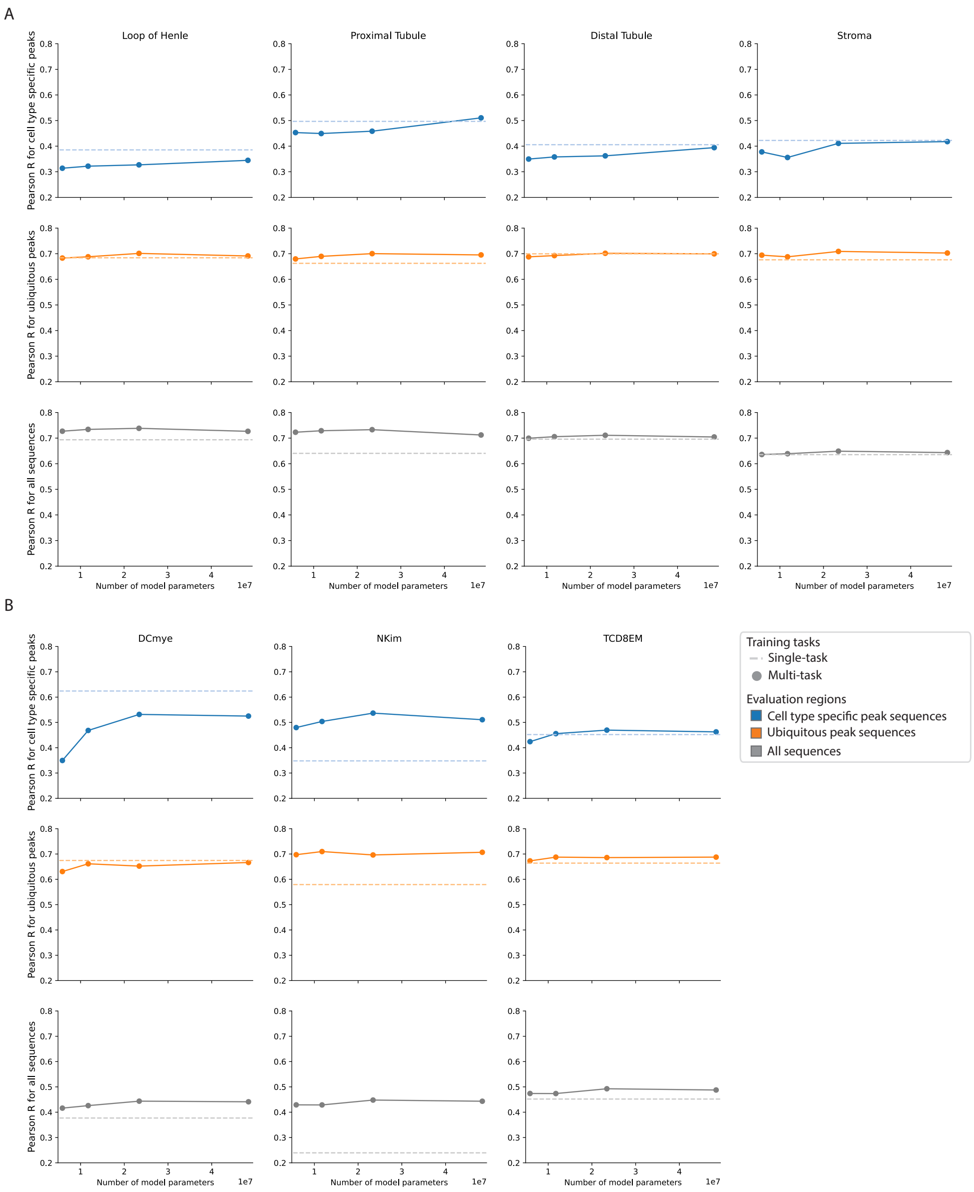

Fig. S15 Increased multi-task model capacity improves cell type specific accessibility prediction. Analysis of the effect of increasing multi-task model capacity on prediction performance in cell type specific peaks, ubiquitous peaks, and all genomic sequences using the A) Loeb et al. [27] and B) Calderon et al. [28] data. For each cell type, performance of a single-task model with a similar number of parameters as the baseline (smallest) multi-task model is shown with a dashed line. Model capacity for the multi-task models was increased by increasing the number of parameters in each layer.

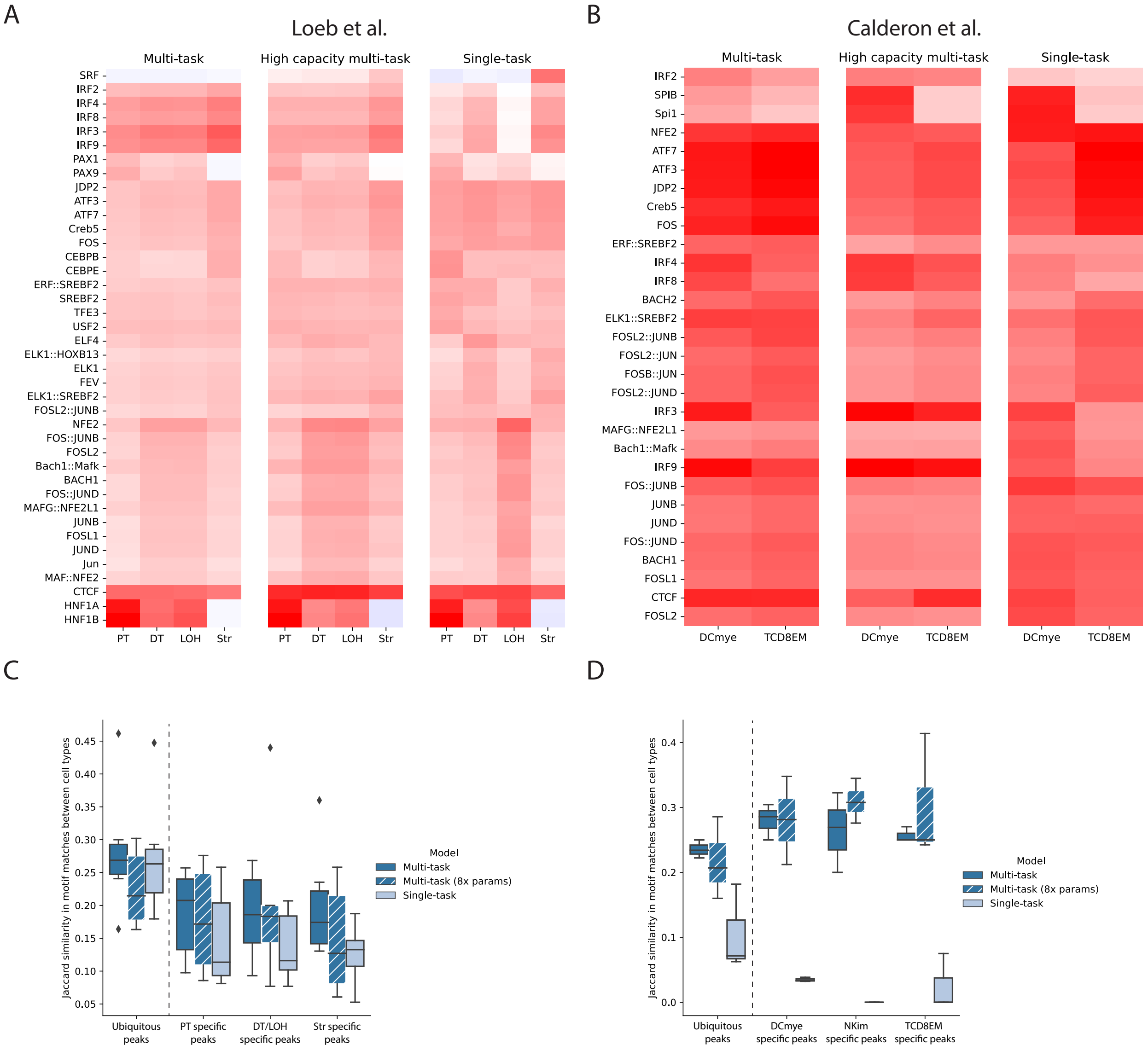

Fig. S16 Interpretability analysis of TF motifs learned by single-task versus multi-task models. We compute model predicted TF activity scores for both the A) Loeb et al. [27] and B) Calderon et al. [28] datasets in each cell type for baseline multi-task, high capacity multi-task, and single-task models. TFs with the greatest model predicted activity (Z-score $\geq$ 3) are plotted. TF activity scores were computed by comparing model predicted activity for a set of dinucleotide shuffled background sequences versus the dinucleotide shuffled background sequences with the TF's canonical motif inserted at the center of the sequence. For both the C) Loeb et al. [27] and D) Calderon et al. [28] datasets, we also use TF-MoDISco to identify the TF motifs driving predictions in ubiquitous and cell type specific peaks. For the baseline multi-task, high capacity multi-task, and single-task models, we report the Jaccard similarity in the motifs identified by TF-MoDISco across cell types.

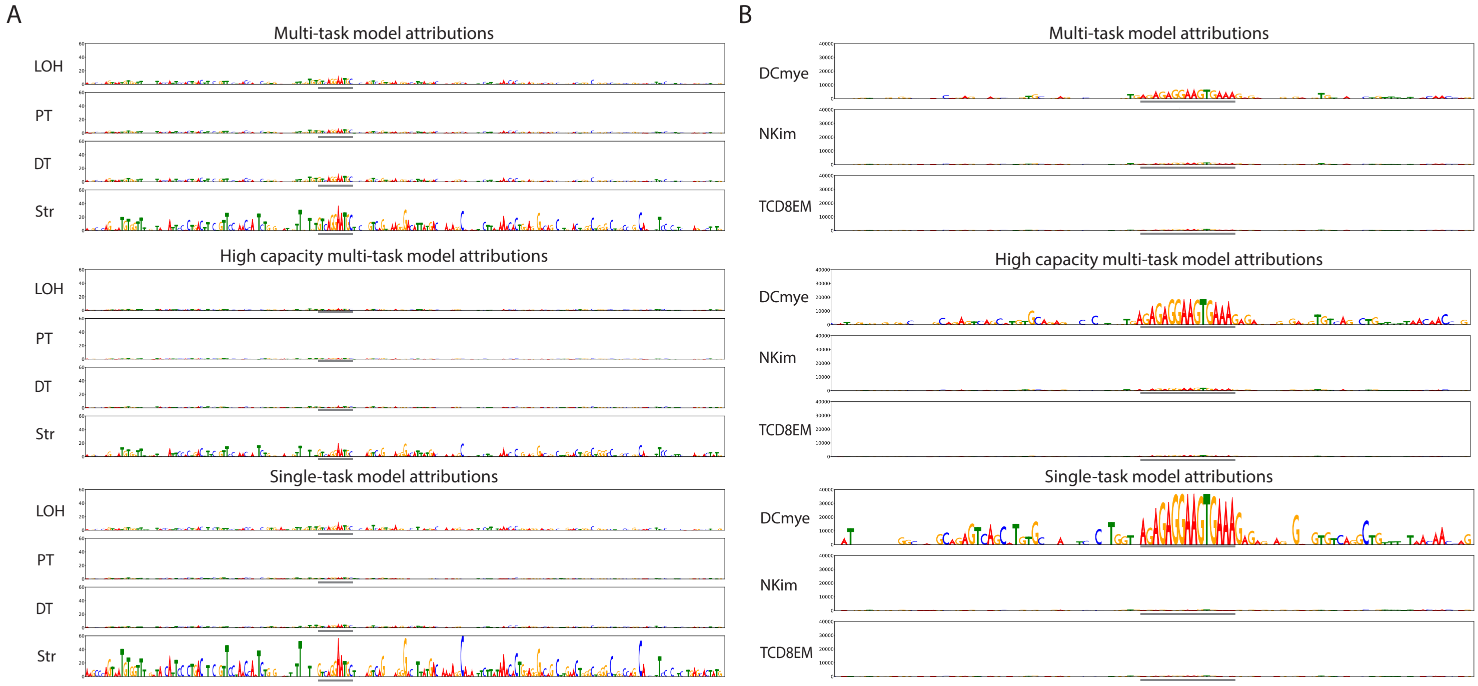

Fig. S17 ***In silico*** **mutagenesis of cell type specific peaks for tissue-specific models.** A) *In silico* mutagenesis (ISM) of a Stromal cell specific peak – chr5:68,137,529-68,137,729 (hg38 coordinates) – that is mispredicted by the baseline multi-task model to be a peak in additional cell types, but is correctly predicted by the single-task models to only be accessible in Stromal cells. The ISM score for each position is the maximum absolute decrease in predicted accessibility over all non-reference nucleotides compared to the reference nucleotide. ISM reveals that a TEAD-like motif is contributing to the predictions of the baseline multi-task model in both Stromal and non-Stromal cell types, while this TEAD-like motif is less apparent in non-Stromal cell types for the high-capacity multi-task and single-task models. The location of the TEAD-like motif is indicated with gray bars underneath the ISM tracks. ISM scores are plotted on the same scale for all models. B) ISM of a dendritic cell specific peak – chr5:73,748,769-73,748,869 (hg19 coordinates) – that is mispredicted by the baseline multi-task model to be a peak in additional cell types, but is correctly predicted by the single-task models to only be accessible in dendritic cells. ISM reveals a SPIB-like motif weakly informing predictions of the baseline multi-task model, and more strongly driving dendritic cell predictions for the high-capacity multi-task and single-task models. The location of the SPIB-like motif is indicated with gray bars underneath the ISM tracks. ISM scores are plotted on the same scale for all models.

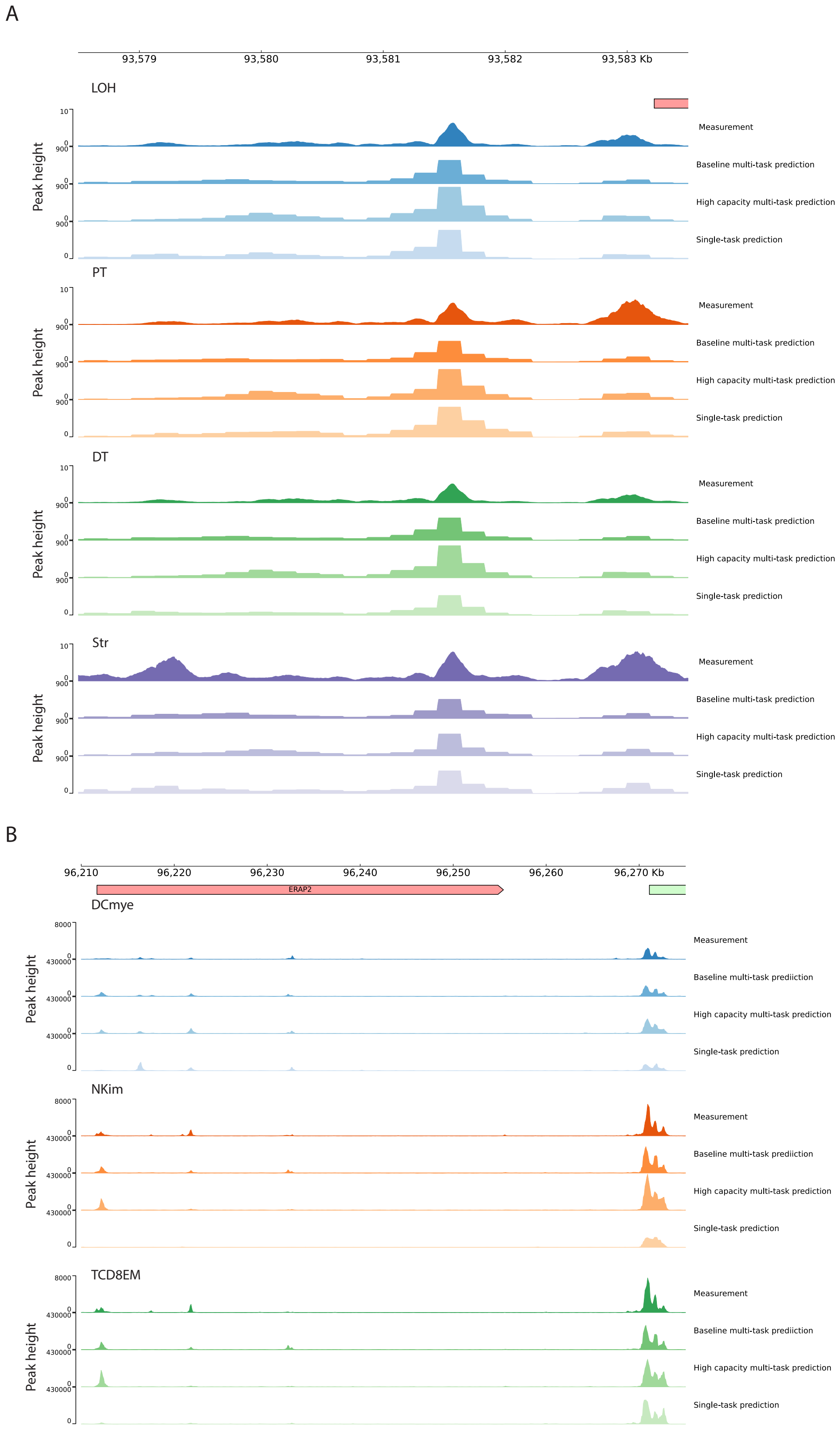

Fig. S18 Single-task versus multi-task model predictions at the *NR2F1* and *ERAP2* loci. A) Experimentally measured accessibility in the Loeb et al. [27] data and predicted accessibility profiles from baseline multi-task, high capacity multi-task, and single-task models for the region around *NR2F1*. B) Experimentally measured accessibility in the Calderon et al. [28] data and predicted accessibility profiles from baseline multi-task, high capacity multi-task, and single-task models for the region around *ERAP2*.

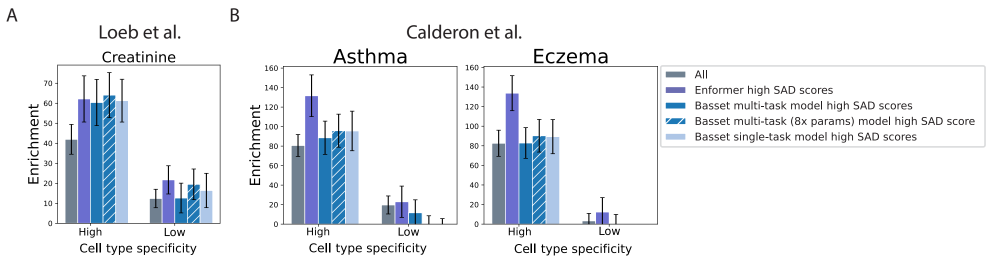

Fig. S19 Comparing variant effect predictions from tissue-specific models and Enformer on GWAS heritability enrichment. For single-task and multi-task models trained on the A) Loeb et al. [27] and B) Calderon et al. [28] data, as well as Enformer, we subset variants in high and low cell type specificity peak regions based on each model's SNP Accessibility Difference (SAD) scores and assess enrichment of trait heritability for tissue-matched traits. We use the median SAD score for all variants in a particular peak set (e.g."Kidney high cell type specificity peaks") as a threshold to subset to high SAD score variants. For Enformer, we use the mean SAD scores across all chromatin accessibility tracks corresponding to the matched tissue (Blood/Immune tracks for Asthma and Eczema; Kidney tracks for Creatinine).
