# Supplementary material for: Current genomic deep learning models display decreased performance in cell type-specific accessible regions
Source: Genome Biol. 2024 Aug 1;25:202. doi: 10.1186/s13059-024-03335-2 (PMC11293111; doi:10.1186/s13059-024-03335-2)
Supplement: Supplementary file 3 — Additional file 3: Peer review history. [file 13059_2024_3335_MOESM3_ESM.docx]

**Review history**

**First round of review**

**Reviewer 1**

Major comments:

1. The prediction performance should be evaluated in a cross-chromosome manner. For example, training the model on chromosomes 2 and above, and evaluating it on chromosome 1, which represents about 10% of held-out data. This guarantees that there is not test-train leakage on the genomic coordinates level. See the paper: https://www.nature.com/articles/s41576-021-00434-9
2. The models were compared in two variations - multi-task and single-task. It would be very interesting to add a 'transfer-learning' approach, where a single-task model is first trained on one cell-type with abundant high-quality data and then fine-tuned to a specific cell type for which less data or of lower quality is available (e.g., https://doi.org/10.1093/nar/gkaa349). We recommend adding this model variant to the comparison.
3. Some of the issues raised in the paper may have been resolved using large DNA language models, such as DNABERT-2 (https://arxiv.org/abs/2306.15006), Grover (https://www.biorxiv.org/content/10.1101/2023.07.19.549677v2.abstract), and GPN (https://www.pnas.org/doi/abs/10.1073/pnas.2311219120). We highly recommend testing their performance, since they may improve performance on cell-type-specific CREs.
4. Some data is unavailable. Reference [22] appears as 'submitted' and there's no link to its data. In addition, the GitHub link does not work. These two issues must be corrected.
5. It would be interesting to see an evaluation and effect of the model choices and training options on the interpretability and not just predictability, as interpretability is a fundamental task in genomics and bioinformatics (e.g., Figure 4c in Enformer paper).

Minor comments

6. Figure 1, caption: cell -> Cell.
7. Figure 2a cannot be referenced after 2b,c. Also, Figure S6, S7 come after S2, and then S4 and S5. Correct all reference order.
8. Results, 1st subsection: Does Sei model outputs a binary value or a probability? (binary value would not allow for ranking, which is required for AUROC, AUPR calculation)
9. Multiple figures: What is the "log-log" Pearson R? we assume it's the Pearson R on the log values of predicted and log values of measured. Thus, it is Pearson R and not "log-log Pearson R".
10. Figure 3e caption - missing ")".
11. Figure S6 legend - there is redundancy which can be resolved by using different ticks.
12. Multiple figures: If bar-plot figures represent 'median' Pearson R, the y-axis title should be "Median Pearson R".
13. Figures 3e 3f - missing y-axis title.
14. Throughout paper: We recommend changing the terms "more" and "less" to "high" and "low", respectively.
15. Discussion: We kindly disagree with the limitation of single-task models. Since we are interested in best prediction, spending a long runtime but only once for training is acceptable to have a model with improved prediction performance.

**Reviewer 2**

Summary: The authors show that the performance of existing models predicting epigenetic features from DNA (Enformer and Sei) within cell type specific CREs is lower compared to ubiquitous CREs for both the "reference accuracy" and for eQTL variant effect prediction. They have also trained multi-task Basset-like models on two much smaller datasets of DNA accessibility for more closely related cell types and have shown that the "reference accuracy" is also lower for these in cell type specific CREs compared to ubiquitously accessible CREs. For these smaller dataset of more closely related cell types, the authors have also trained single task models which showed higher "reference accuracy" in cell type specific CREs, although the performance was still significantly lower compared to ubiquitous CREs and they haven't really shown consistent improvement in variant effect prediction accuracy compared to multi-task models.

Overall, I find the manuscript interesting and novel. I expect it to provide helpful insights to the research community of model developers aiming to better understand the cis regulatory code using deep learning models. However, I don't think the manuscript in its current form is ready for publication. I have outlined my major and minor points that I think need to be addressed below.

Major:

1. The authors use CRE and DNA accessibility peaks interchangeably. Not all DNA accessibility peaks are cis regulatory elements. Therefore, the authors should be much more explicit about this. This might require replacing most/all mentions of CRE with DHS peaks or use candidate CREs defined by the ENCODE consortium for their analysis.
2. This manuscript doesn't provide direct insights into how to improve variant effect prediction accuracy which is the main application of models predicting chromatin features from DNA sequence. The authors have not demonstrated that single-task training leads to better variant effect prediction. Only in one cell type (Stroma in Fig. S6) out of 7 shown in Fig. S6 and S7 is single task training performing convincingly better in variant effect prediction compared to multi-task learning. Variant effect prediction is the most important task for these models and the authors acknowledge that by saying the performance shouldn't be just measured by 'reference accuracy' since it is unclear how this will translate to variant effect prediction accuracy in the main text. In the abstract, however, the authors say that they "identify single-task learning as a potential method to improve performance in cell type specific CREs.". Combining both makes the implicit argument that single task learning will lead to better variant effect prediction, which is not supported. The authors should make that limitation explicit in the abstract and at numerous points in the manuscript including at L331-L348.
3. Abstract: "we find that the accuracy of two state-of-the-art general purpose models… varies dramatically across the genome" -> The authors find this also for single task models, not just for general purpose models. This sentence implies as if only the general purpose models have this limitation which is misleading. The single task models only close a small performance gap between cell type specific vs ubiquitous CREs and therefore suffer from the same issues.
4. The authors don't evaluate single task models on GWAS or eQTL benchmarks nor do they directly compare them to existing models. Therefore, the authors should also re-train the single-task and multi-task models on either Sei or Enformer data sets similarly to what they have done for Loeb/Calderon data and evaluate the performance on eQTL causality and directionality benchmarks.
5. Multi-task vs single-task model comparison: The authors should make sure that the model capacity (say number of layers or channels) is appropriately larger for the multi-task model. Currently, the same hyper-parameters are used for both models which may lead to sub-optimal performance of either the multi-task or the single-task models.
6. The manuscript would also benefit from more in depth analysis of contribution scores (computed using say in silico mutagenesis or input*gradient) comparing single task models vs multi-task models when looking at cell-type specific CREs and/or variant effect prediction.
7. The flow of the manuscript could be improved. There are a number of occasions where the order of figures is inconsistent with when they are mentioned in the text and also some important details are not mentioned such as that a multi-task model was shown in Fig. 3. I've tried to flag a few of these instances in minor points below.
8. L353: "similar task groupings may be a promising approach to combine performance improvements" - what kind of task groupings have the authors in mind here? Grouping similar cell types (as shown in Fig. 3 which also performs sub-optimally) or grouping different assays measuring the same cell type?
9. https://github.com/nilab/CellTypeSpecificAccessibilityPrediction code is not accessible. Could the authors please share the code as well? I would expect this repository to allow researchers to easily re-evaluate new models on the benchmarks posed by the manuscript.

Minor:
1. L71 "accessibility characterizes active CREs,". Accessible CREs are not necessarily transcriptionally active. Would therefore suggest saying 'potentially active CRE'
2. L99: "...provide a comprehensive view of the performance of current state-of-the-art genomic deep learning models in CREs" - The evaluation doesn't consider comparison to say MPRA data which means that it's not comprehensive. The authors should therefore remove the "comprehensive" claim.
3. Fig. 2A: The authors should also attempt to add the performance of experimental replicates. Some of the peaks accessible only in a single cell type might be technical artifacts. If experimental replicates are not available, then the authors should use the most similar track as a substitute for the experimental replicate.
4. Fig. 2A: When quantifying the cell type specificity of predictions, the authors should also look at alternative metrics such as precision per CRE for the top N predictions, where N is the number of accessible cell types for that cell type. Note that one should z-score normalize the predictions for each predicted experiment first before doing this comparison. Another relevant evaluation metric would be to look at how well the models can predict differentially accessible regions given a specific pair of cell types.
5. Fig. 2B: How much of that figure can be explained by distance from TSS or element types (promoter vs enhancer)?
6. Fig. 2C is introduced before Fig. 2a (L123 vs L129)
7. Fig. 2D should also contain the bar showing the enrichment from 2C for easier comparison to all/non-stratified variants using Enformer. Figure legend should also mention 'Low/high Enformer SAD scores'. The authors should also mention that using variants in more cell type specific peaks with high Enformer SAD score are even more highly enriched for trait heritability than variants in cell type specific peaks generally.
8. L179: "Taken together, …these results indicate… that models have decreased … accuracy." -> I don't think Fig. 2D is showing lower performance in cell type specific CREs. Therefore, I would suggest moving the Fig. 2C and D to a separate section with its own conclusion sentence.
9. Fig. S3A y-axis should be shown in log-scale.
10. L87: "...we reasoned that negative transfer led to sub-optimal performance": There might be other reasons beyond negative transfer for multi-task models to not be performing as well such as limited capacity. Have the authors carefully re-trained the multi-task vs single-task models while matching for the right model capacity to demonstrate the impact of negative transfer?
11. Fig. 3A: Why is the y-axis in different units (proportion heritability) vs Fig. 2C,D (enrichment).
12. L212: "All models achieve a genome-wide reference accuracy comparable to previous work (Fig. S6, Fig. S7)". Which of the curves in Fig. S6, S7 is showing previous work?
13. Figures S6, S7 are introduced before Fig. S4 in the text. Also, single-task models haven't been mentioned until then.
14. L208: Do the authors train only single-task models or multi-task models here?
15. Fig. 3: Is the model compared here multi-task or single-task? I've had trouble figuring this out from the figure legend / text.
16. Fig. 3C vs 4A -> consistent colors should be used for multi-task prediction models since they are showing the same performance numbers or?
17. L222: "...model trained on related cell types also have decreased performance…" - what do the authors mean by 'trained on related cell types'? Weren't the models trained in the same cell type as they are being evaluated on? -> After re-reading the text a few times I realized the text was actually talking about a multi-tasking model. The authors should make that more explicit in the text and include 'multi-task' in the title of this section.
18. Fig. 3E,F -> The authors should also show the same prediction example for the single-task models discussed in Fig. 4.
19. Fig. S6, S7 are very difficult to read. The authors should consider splitting these out into multiple panels.

**Reviewer 3**

In this manuscript, the authors identified a limitation in current genomic deep learning models: the accuracy of these models decreases in cell type-specific CREs. This includes both prediction accuracy for the reference sequence and for variant effects. The authors then compare single-task models to multi-task models and report an improvement in cell-type-specific CRE predictions. Overall, this is a useful benchmark study that contributes to our understanding of genomic deep learning model performance. However, I have the following concerns:

Major points:

1. The authors observed an over-correlation between model predictions for different cell types compared to experimental measurements. While it is plausible that this over-correlation is partially due to model bias, another potential source of over-correlation is experimental measurement noise. With noisy experimental measurements, the correlation between cell types is expected to be lower than between predictions, even if the predictions are unbiased. Therefore, over-correlation should not be simply considered as a bias of the models. The effect of experimental noise should ideally be taken into account in the analysis.

2. The comparison between single-task and multi-task models is informative, but the results are not entirely conclusive in my opinion. The improvement of single-task models in cell type-specific CRE predictions is mild and inconsistent across all cell types. Additionally, the effect on variant effect prediction in cell type-specific CREs is mixed, as shown in Figs. S6b and S7b. The performance of single-task models on ubiquitous peaks seems to be similar or slightly worse overall. Although the single-task model demonstrates a partial reduction in over-correlation compared to the multi-task model, it is unclear whether this reduction is an improvement or a result of increased variance across single-task models. Moreover, the influence of the number of parameters on the results warrants investigation. It might be worthwhile to explore scenarios where the sum of all single-task model parameters is equal to that of the multi-task model, and where each individual single-task model has the same number of parameters as the multi-task model.

Minor points:

1. The figure panels are not ordered in the same order as they are mentioned in the text. For example, Figure 2c is referenced before Figure 2a in the text.
2. One of the two datasets primarily used in the experiments (Loeb et al.) is not yet publicly available.
3. The models trained on Calderon et al. and Loeb et al. are not available.

**Reviewer 4**

Review: Current genomic deep learning models display decreased performance in cell type specific cis regulatory elements


The manuscript's premise is that DNA sequence-based multi-task deep learning (DL) predictors generally focus on genome-wide accuracy. However since disease heritability is enriched in CREs, it is important to assess whether these models are equally accurate in CREs with varying degrees of cell-type specificity. Here, the authors assess the performance of two multitask models (Sei and Enformer) at predicting cell-type specific accessibility and conclude that both models display decreased performance over cell-type specific CREs when compared to shared / ubiquitous CREs.

This is an interesting perspective and I believe that this an important assessment to perform as we begin utilizing sequence-based DL models to evaluate genetic variation. However, in its current form, the evidence provided in order to reach the stated conclusions is insufficient and a more complete analysis would make this research more useful.

Concerns:

1. The first major claim in the paper is that cell-type specific accessibility peaks are more enriched for heritability when compared to shared CREs ( Fig. 2C, D). This conclusion is based on stratified LD score regression (S-LDSC) which partitions heritability given a set of annotations. The authors perform S-LDSC by including the baseline LD annotation set. The baseline LD annotations contain several annotations that would overlap extensively with shared CRE features but not cell-type specific CREs - such as FANTOM 5 enhancers, promoters etc. Since the heritability estimates provided by S-LDSC are conditional estimates, it is unclear whether the decreased heritability in shared CREs is an artifact driven by the inclusion of other annotations marking these regions. Performing S-LDSC without conditioning on noncoding baseline annotations would provide an unbiased estimate of heritability enrichment in shared vs. cell-type specific CREs.

2. In Fig. 2D, loci are divided into "high" vs. "low" magnitude Enformer variant scores and heritability enrichment is computed for each category. How is a threshold chosen to divide predictions into categories? Computing statistical significance & computing heritability enrichment for significant vs. non-significant variant effects would be more convincing than using a single threshold to categorize variant effects.

3. In Fig. 3A, the author's measure the proportion of heritability within cell-type specific vs. shared CREs for the two ATAC-seq datasets. However, the proportion of heritability will be higher for a category if it contains a greater proportion of SNPs. It would be nice to see heritability enrichment reported here (controlling for number of SNPs in annotation). This would also be consistent with Fig. 2C, which already reports heritability enrichment.

4. In Fig. 3C, accuracies over cell-type specific CREs are reported for 4 and 3 cell-types from the Loeb at al. and Calderon et al. datasets shown. For a more complete evaluation, it would be nice to see the results for all cell-types, this could be added to supplementary figures.

5. Figure 3D: While assessing prediction uncertainty, is the coefficient of variation (uncertainty in prediction) significantly different between cell-type specific vs. shared CREs, especially in the Calderon et al. set?

6. The regulatory grammar at TSSs is distinct from that at distal enhancers - is the model's ability to learn cell-type specificity dependent on whether the CRE is a proximal element such as a TSS or distal enhancer?

7. For specific peaks which are mis-predicted, are false negatives or false positives the problem? If these peaks are false positives, what motifs are driving the predictions? To assess this, analyzing model attributions would be useful.

8. Why is cell type specific performance worse? The authors seem to favor model bias, but several alternatives are worth considering:
8a. Ubiquitous peaks have a greater true positive rate because specific peaks are more likely false positive peak calls or attributable to genetic variation outside the reference. Authors could assess using ENCODE replicates whether ubiquitous peaks are more likely to appear in multiple replicates.
8b. Do ubiquitous peaks have greater information content than specific peaks? I.e. CpG island promoters and CTCF insulators are very easy to predict. Are specific peaks more likely to be driven by complex grammars that are inherently more challenging for ML models to learn?

Additional Minor Concerns:
Figure 1A & 1B: Number of cell-types reported on the x-axis is incorrect. There are 684 DNase tracks in the Enformer/ but not all of them are distinct cell-types. For example, there might be several K562 tracks from distinct sources. Similarly, the legend is also incorrect in S1A and S1B.

**Authors’ response to reviewers**

We thank the reviewers for their thoughtful comments and suggestions, which have considerably strengthened our manuscript. We have carried out several additional analyses and revised the manuscript to strengthen the conclusions and address their concerns. The new analyses include a new main figure panel (Figure 4c), 13 new supplementary figures (Figures S1, S3, S4, S6, S8, S10, S13-19), and 1 new supplementary table (Supplementary Table 1).

We highlight two notable ways we have improved the manuscript:

1) We have performed a more thorough comparison of single-task versus multi-task learning to disentangle the respective roles of increased model capacity versus negative transfer in the performance improvements seen for single-task models within cell type specific accessible regions. The full results of this analysis are shown in Figure S15, and we have also included results from the highest capacity multi-task model in Figures 4 and S9. We find that increased capacity multi-task models tend to improve predictive accuracy in cell type specific peaks, in many cases approaching the performance of single task models (Fig. 4a). These results provide evidence that the benefit we observe from single-task models may largely be due to increased model capacity, which may give the model more “room” to learn cell type specific regulatory syntax. Depending on the particular use case and computational budget, single-task models or higher capacity multi-task models may both be ways to achieve this. We have modified the conclusions we draw from the single-task versus multi-task comparison to reflect this information, and more generally recommend increasing model capacity to improve cell type specific performance.
2) We have clarified throughout the manuscript whether the evaluated training decisions improve reference accuracy versus variant effect accuracy. In particular, we have more clearly delineated in both our results and discussion that although single-task models improve cell type specific reference accuracy, they do not consistently improve variant effect accuracy. We also believe this is a valuable finding that improvements in reference sequence prediction do not always lead to direct improvement in variant effect prediction, indicating that novel training strategies may be needed to improve variant effect accuracy. We have added text to this effect in our discussion.

In addition to the changes described above, we have made several other additions and modifications to the manuscript based on the reviewers’ suggestions. Our point-by-point response is below.

# Reviewer #1:

Major comments:

1. The prediction performance should be evaluated in a cross-chromosome manner. For example, training the model on chromosomes 2 and above, and evaluating it on chromosome 1, which represents about 10% of held-out data. This guarantees that there is not test-train leakage on the genomic coordinates level. See the paper: https://[www.nature.com/articles/s41576-021-00434-9](http://www.nature.com/articles/s41576-021-00434-9)

We agree that prediction performance should be evaluated in a cross-chromosome manner and had already implemented this strategy for the kidney and immune tissue specific models that we used to evaluate different training paradigms (i.e. single versus multi-task). In particular, we used chromosomes 7, 14, and 15 as a validation set, and held out chromosomes 4 and 5 for evaluation. For these models, the prediction performance reported throughout the paper is on the held out chromosomes. We have added additional text in the relevant results sections to make the evaluation strategy more clear. For example, in the Figure 3 caption, we added the following text: “Plotted points are sequences from the held out test chromosomes.”

For the two additional models that we evaluated – Enformer and Sei – we used the pre-trained versions of the models provided by the authors in their publications. In these cases, the prediction performance we report is on the sequences held out during training by the authors of each model. For Enformer, sequences were not held out in a cross-chromosome manner.

Instead, because the training data consisted of both human and mouse sequences, the authors attempted to minimize leakage of orthologous sequences between the training and test sets (procedure described here). For Sei, chromosome 10 was used for validation and chromosomes 8 and 9 were held out for evaluation.

2. The models were compared in two variations - multi-task and single-task. It would be very interesting to add a 'transfer-learning' approach, where a single-task model is first trained on one cell-type with abundant high-quality data and then fine-tuned to a specific cell type for which less data or of lower quality is available (e.g., https://doi.org/10.1093/nar/gkaa349). We recommend adding this model variant to the comparison.

We thank the reviewer for this interesting suggestion. We have performed the following test to evaluate the suggested transfer learning approach, and have added the results as a supplementary figure (Fig. S13). Using the Loeb, et al. data, we trained two additional model variants using a downsampled version of the Proximal Tubule data (downsampled to approximately 10% of the original number of collected cells): 1) a single task model trained from scratch on the downsampled Proximal Tubule data, and 2) a single task model pretrained on the Stromal cell data and then fine-tuned on the downsampled Proximal Tubule data. We then evaluated how well each of these models was able to predict Proximal Tubule specific peaks, ubiquitous peaks, and all genomic sequences (peaks and non-peaks). We find that the transfer learning procedure decreases predictive accuracy on cell type specific peaks, and leads to a small increase in accuracy on ubiquitous peaks. This result complements our existing single- task versus multi-task comparisons, which suggest that cross cell type training does not provide

a benefit for cell type specific peak prediction. There may be other cases – for example, when the pretraining and target cell types are more closely related, or when the data for the target cell type is even more sparse – where this transfer learning paradigm may provide some utility, which would be interesting avenues for future exploration.

3. Some of the issues raised in the paper may have been resolved using large DNA language models, such as DNABERT-2 (https://arxiv.org/abs/2306.15006), Grover (https://[www.biorxiv.org/content/10.1101/2023.07.19.549677v2.abstract),](http://www.biorxiv.org/content/10.1101/2023.07.19.549677v2.abstract)) and GPN (https://[www.pnas.org/doi/abs/10.1073/pnas.2311219120).](http://www.pnas.org/doi/abs/10.1073/pnas.2311219120)) We highly recommend testing their performance, since they may improve performance on cell-type-specific CREs.

We agree with the reviewer that DNA language models are a promising direction that might overcome some of the issues faced by supervised genomic deep learning models, and we think this is a fascinating area for future exploration. Because DNA language models are trained in an unsupervised manner using only DNA sequences, without any cell type specific data, their zero- shot predictions are by nature cell-type agnostic. We conducted a preliminary comparison of DNABERT-2, Enformer, and Sei on the eQTL classification task presented in Fig. 2C and observe that zero-shot predictions from DNABERT-2 have minimal signal for classifying fine- mapped eQTLs (figure below). However, fine-tuning language models such as DNABERT-2 on functional genomics data, such as cell-type specific accessibility data, may yield better performance. Such fine-tuning will be an interesting avenue for future exploration, but is beyond the scope of our current work. We believe the primary value of our current work is in evaluating supervised models, which (1) are becoming more routinely adopted for a variety of variant effect prediction and genome interpretation tasks, and (2) by virtue of their training procedure, naturally make cell-type specific predictions without the need for any additional training or fine- tuning.

4. Some data is unavailable. Reference [22] appears as 'submitted' and there's no link to its data. In addition, the GitHub link does not work. These two issues must be corrected.

We apologize that there was a typo in the Github link in our original manuscript. We have fixed this error, and the Github can be accessed here: https://github.com/ni- lab/CellTypeSpecificAccessibilityPrediction. The data from reference [22] have also been deposited to GEO; we will share the link to the data on GEO as soon as it is available (within 5 days of GEO submission). In addition, reference [22] is in the final stages of review and we will ensure that it is publicly available before publication of this manuscript.

5. It would be interesting to see an evaluation and effect of the model choices and training options on the interpretability and not just predictability, as interpretability is a fundamental task in genomics and bioinformatics (e.g., Figure 4c in Enformer paper).

We agree that interpretability is an important task and use case for genomic deep learning models. We have performed two additional analyses to illustrate the effect of single-task vs. multi-task training on interpretability: a global interpretability analysis and a local interpretability analysis, which we have incorporated into the paper in Supplementary Figures 16 and 17.

Global interpretability analysis

We reasoned that differences in cell-type specific prediction between models are likely to be driven by differences in their ability to recognize cell-type specific TF motifs and to correctly associate these motifs with accessibility in particular cell types. To assess this, we performed a motif insertion analysis using both the single-task and multi-task models (Fig. S16a,b). We computed TF activity scores for each TF in each cell type and model by comparing predicted activity for a set of dinucleotide shuffled background sequences versus background sequences with the TF’s canonical motif inserted. In both the Loeb et al. and Calderon et al. datasets, we visually observe greater heterogeneity in model-predicted TF activity scores across cell types for the single-task models as compared to the multi-task model. This suggests that some of the over-correlation between cell types that we observe for the multi-task models (Fig. 4b) may be due to the models learning a similar regulatory syntax for distinct cell types.

We note some particular cases where the single-task models have learned cell-type specific TF activity that is missed by the multitask model. For example, in the Loeb et al. data, the single- task models correctly identify SRF as a Stromal specific TF, while the multitask model fails to learn this TF as active in any cell type (Fig. S16a). In the Calderon et al. data, the single-task models identify SPI1 as a myeloid specific TF, while the multitask model instead predicts only weak SPI1 activity across cell types (Fig. S16b). Similarly, the single-task models predict more specific TF activity for FOS/JUN TFs (FOSL2::JUNB, FOSL2::JUN, FOSB::JUN, FOSL2::JUND)

in CD8 T cells, while the multitask model predicts these TFs to be broadly active across the cell types (Fig. S16b).

Local interpretability analysis

We also performed a local interpretability analysis to highlight specific examples of the motifs that are driving the predictions of the single-task and multi-task models.

In the Loeb, et al. data, we highlight a Stromal cell specific peak that is mispredicted by the multitask model to be a peak in the other cell types as well, but is correctly predicted by the single-task models to only be accessible in Stromal cells. *In silico* mutagenesis of this peak reveals that a TEAD-like motif is driving the prediction of the multi-task model in Stromal and non-Stromal cell types (Fig. S17a). By contrast, this TEAD-like motif is less apparent in the single-task models trained on the non-Stromal cell types.

In the Calderon, et al. data, we highlight a dendritic cell specific peak that is mispredicted by the multitask model to be a peak in the other cell types as well, but is correctly predicted by the single-task models to only be accessible in dendritic cells. *In silico* mutagenesis of this peak reveals that a SPIB-like motif is weakly driving the prediction of the multi-task model in dendritic and non-dendritic cell types (Fig. S17b). By contrast, this SPIB-like motif is only driving predictions for the dendritic cell single-task model. Notably, the dendritic cell single-task model also predicts a much stronger effect of the SPIB-like motif, mirroring the differences in predicted TF activity in Fig. S16b, where we observed stronger and more specific predicted SPIB activity in dendritic cells with single-task models.

Minor comments

6. Figure 1, caption: cell -> Cell. We have fixed this typo.
7. Figure 2a cannot be referenced after 2b,c. Also, Figure S6, S7 come after S2, and then S4 and S5. Correct all reference order.

Thank you for pointing this out; we have corrected the reference orderings.

8. Results, 1st subsection: Does Sei model outputs a binary value or a probability? (binary value would not allow for ranking, which is required for AUROC, AUPR calculation)

The Sei model outputs a probability. We have updated the relevant results section to read: “For the Sei model, which predicts a probability of ~~binary value corresponding to~~ the presence ~~or~~ ~~absence~~ of a peak, we report the AUC and AUPRC for the predictions in each bin.”

9. Multiple figures: What is the "log-log" Pearson R? we assume it's the Pearson R on the log values of predicted and log values of measured. Thus, it is Pearson R and not "log-log Pearson R".

You are correct that we are referring to the Pearson R on the log predicted and log measured values. We have updated the text and figures to read “Pearson R” to avoid confusion.

10. Figure 3e caption - missing ")". We have fixed this typo.
11. Figure S6 legend - there is redundancy which can be resolved by using different ticks. We have updated the formatting of the previous Figures S6 and S7 (now Figures S11 and S12) for clarity. In particular, we have taken the reviewer’s suggestion to use different ticks to limit

redundancy in the figure legend. In addition, we have split out the results for each set of evaluation regions – cell type specific peaks, ubiquitous peaks, and all sequences – into separate figure panels for readability, as suggested by another reviewer.

12. Multiple figures: If bar-plot figures represent 'median' Pearson R, the y-axis title should be "Median Pearson R".

We have updated the axis titles to “Median Pearson R.”

13. Figures 3e 3f - missing y-axis title. We have fixed this.
14. Throughout paper: We recommend changing the terms "more" and "less" to "high" and "low", respectively.

We thank the reviewer for this suggestion. We have modified the language throughout the paper to refer to “high” and “low” cell type specificity peaks for clarity.

15. Discussion: We kindly disagree with the limitation of single-task models. Since we are interested in best prediction, spending a long runtime but only once for training is acceptable to have a model with improved prediction performance.

We thank the reviewer for their perspective on this point. We have removed the discussion sentence about training time as a limitation of single-task models.

# Reviewer #2:

Summary: The authors show that the performance of existing models predicting epigenetic features from DNA (Enformer and Sei) within cell type specific CREs is lower compared to ubiquitous CREs for both the "reference accuracy" and for eQTL variant effect prediction. They have also trained multi-task Basset-like models on two much smaller datasets of DNA accessibility for more closely related cell types and have shown that the "reference accuracy" is also lower for these in cell type specific CREs compared to ubiquitously accessible CREs. For these smaller dataset of more closely related cell types, the authors have also trained single task models which showed higher "reference accuracy" in cell type specific CREs, although the performance was still significantly lower compared to ubiquitous CREs and they haven't really shown consistent improvement in variant effect prediction accuracy compared to multi-task models.

Overall, I find the manuscript interesting and novel. I expect it to provide helpful insights to the research community of model developers aiming to better understand the cis regulatory code using deep learning models. However, I don't think the manuscript in its current form is ready for publication. I have outlined my major and minor points that I think need to be addressed below.

Major:

1. The authors use CRE and DNA accessibility peaks interchangeably. Not all DNA accessibility peaks are cis regulatory elements. Therefore, the authors should be much more explicit about this. This might require replacing most/all mentions of CRE with DHS peaks or use candidate CREs defined by the ENCODE consortium for their analysis.

We appreciate the critique that “CRE” and “DNA accessibility peaks” should not be used interchangeably because not all DNA accessibility peaks are CREs. We have replaced most mentions of CREs throughout the paper (except when explicitly referring to candidate *cis* regulatory elements) with “chromatin accessibility peaks.”

2. This manuscript doesn't provide direct insights into how to improve variant effect prediction accuracy which is the main application of models predicting chromatin features from DNA sequence. The authors have not demonstrated that single-task training leads to better variant effect prediction. Only in one cell type (Stroma in Fig. S6) out of 7 shown in Fig. S6 and S7 is single task training performing convincingly better in variant effect prediction compared to multi- task learning. Variant effect prediction is the most important task for these models and the authors acknowledge that by saying the performance shouldn't be just measured by 'reference accuracy' since it is unclear how this will translate to variant effect prediction accuracy in the main text. In the abstract, however, the authors say that they "identify single-task learning as a potential method to improve performance in cell type specific CREs.". Combining both makes the implicit argument that single task

learning will lead to better variant effect prediction, which is not supported. The authors should make that limitation explicit in the abstract and at numerous points in the manuscript including at L331-L348.

We completely agree with the reviewer’s observation that single-task models don’t consistently improve variant effect prediction. We have modified our text throughout the manuscript to clarify which results refer to reference accuracy versus variant effect accuracy, and to more clearly convey our findings that improvements in cell type specific peak “reference accuracy” do not necessarily translate to improvements in variant effect accuracy. We believe that our results identifying modeling strategies that improve reference sequence prediction but not variant effect prediction provide an important insight – namely, that these two evaluation metrics are not always correlated – to help guide future modeling strategies and evaluations emphasizing variant effect accuracy. We have also added text to this effect in our discussion.

3. Abstract: "we find that the accuracy of two state-of-the-art general purpose models… varies dramatically across the genome" -> The authors find this also for single task models, not just for general purpose models. This sentence implies as if only the general purpose models have this limitation which is misleading. The single task models only close a small performance gap between cell type specific vs ubiquitous CREs and therefore suffer from the same issues.

We appreciate that this wording was confusing and have modified the relevant sentence in the Abstract to clarify that this finding is not unique to general purpose models: “We find that the accuracy of genomic deep learning models, including two state-of-the-art general purpose models – Enformer and Sei – varies dramatically across the genome...”

4. The authors don't evaluate single task models on GWAS or eQTL benchmarks nor do they directly compare them to existing models. Therefore, the authors should also re-train the single- task and multi-task models on either Sei or Enformer data sets similarly to what they have done for Loeb/Calderon data and evaluate the performance on eQTL causality and directionality benchmarks.

We have added an additional panel to Figure 4 (Fig. 4c), as well as a supplementary figure (Fig. S19), evaluating the tissue-specific single-task and multi-task models on GWAS benchmarks. (We used the GWAS, rather than eQTL, benchmark due to the limited sample size of the finemapped GTEx eQTL set for kidney tissue.) We find that high SAD score variants from the single-task models and the baseline multi-task models are similarly enriched for trait heritability (Fig. 4c), consistent with the finding noted above that single-task models do not consistently outperform multi-task models on variant effect prediction tasks.

We also compare these enrichments to those for variants with high Enformer SAD scores (for matched tissue tracks), and we observe quite different results for the kidney-related trait (Creatinine) versus the immune-related traits (Asthma and Eczema). For Creatinine, high SAD scores from the single-task and multi-task versions of the tissue-specific models have similar enrichment to high SAD scores from Enformer (Fig. S19a). However, for the immune-related

traits, Enformer’s high SAD scores are significantly more enriched for trait heritability than high SAD scores from the tissue-specific models (Fig. S19b). Differences in performance between the Basset models and Enformer could be due to several factors, such as training on a larger number of cell types, tissues, and assays (i.e. CAGE-seq and ChIP-seq), as well as a higher capacity model architecture. (Note that we do not compare to single-task versions of Enformer due to the computational cost of training the Enformer model.) One possible explanation for the differences between immune and kidney traits is the relative makeup of different track types in the Enformer training data. In particular, immune cell types and tissue samples are heavily overrepresented, which may bias Enformer towards learning immune cell regulatory syntax.

Although this observation warrants further exploration for additional tissues and traits in future studies, it is interesting that variant effect predictions from a significantly smaller model trained on much less data appear to perform on par with Enformer for some traits.

5. Multi-task vs single-task model comparison: The authors should make sure that the model capacity (say number of layers or channels) is appropriately larger for the multi-task model. Currently, the same hyper-parameters are used for both models which may lead to sub-optimal performance of either the multi-task or the single-task models.

We thank the reviewer for this suggestion and agree that model capacity is an important consideration in fairly evaluating the multi-task versus single-task modeling paradigm. We initially used the same model architecture and hyperparameters for single-task and multi-task models, meaning that the single-task models have more capacity (i.e. parameters) to learn about each particular cell type. We have added an experiment to help disentangle the respective roles of increased model capacity versus negative transfer in the performance improvements seen for single-task models. Specifically, for both the Loeb, et al. and Calderon, et al. datasets, we have trained three additional models with 2, 4, and 8 times the number of parameters of the original multi-task models. (Training larger and larger multi-task models quickly becomes computationally prohibitive.) We then evaluated performance of these progressively larger multi-task models in different peak regions, as done throughout the paper. These results are shown in a new supplementary figure (Fig. S15). We have also included results from the 8x capacity multi-task models in the relevant comparisons in Fig. 4 and Fig. S9. We find that when we measure predictive accuracy genome-wide or in ubiquitous peaks, we see little to no improvement from increasing model capacity (Fig. S15a,b, in gray and orange, respectively). However, in cell type specific peaks, we do indeed see that increasing model capacity improves predictive accuracy, in many cases approaching the performance of single task models (Fig. 4, in hatched blue; Fig. S15a,b, in blue). These results provide evidence that the benefit we observe from single-task models may largely be due to increased model capacity, which potentially gives the model more “room” to learn a potentially long tail of cell type specific TFs. Depending on the particular use case and computational budget, single-task models or higher capacity multi-task models may both be ways to achieve improved performance in cell type specific regions. We have modified the conclusions we draw from the single-task versus multi-task comparison to reflect this information, and more generally recommend increasing model capacity to improve cell type specific performance.

6. The manuscript would also benefit from more in depth analysis of contribution scores (computed using say in silico mutagenesis or input*gradient) comparing single task models vs multi-task models when looking at cell-type specific CREs and/or variant effect prediction.

We appreciate this suggestion, which is similar to a suggestion from Reviewer #1. We have performed two additional analyses to explore the effect of single-task vs. multi-task training on model interpretability and contribution scores: a global interpretability analysis and a local interpretability analysis (new Figures S16 and S17). Please see the above response to Reviewer #1, comment 5 for more details.

7. The flow of the manuscript could be improved. There are a number of occasions where the order of figures is inconsistent with when they are mentioned in the text and also some important details are not mentioned such as that a multi-task model was shown in Fig. 3. I've tried to flag a few of these instances in minor points below.

We appreciate this feedback and have made considerable effort to improve the flow of the manuscript, including ensuring that the order of figures is consistent with the order they are mentioned in the text, as well as making clear which models (i.e. single-task vs. multi-task) are being used in each specific evaluation.

8. L353: "similar task groupings may be a promising approach to combine performance improvements" - what kind of task groupings have the authors in mind here? Grouping similar cell types (as shown in Fig. 3 which also performs sub-optimally) or grouping different assays measuring the same cell type?

The task groupings we had in mind were to group the most highly related cell types even within a tissue; for example, of the 10 cell types in the Loeb, et al. kidney data, 5 of these are tubule cell types that share significant biology, while the other 5 are non-epithelial cell types such as immune cells. As a preliminary test of this hypothesis, we trained an additional multi-task model over only the 5 tubule cell types in the Loeb, et al. data. However, we observe that the cell-type specific performance of this new model is only slightly better than the full multi-task model (figure below). Although there are other approaches that could be explored in the future, such as hierarchically grouping cell types, we have removed the sentence quoted by the reviewer from the discussion, as we do not believe there is currently enough evidence to support this direction.

9. https://github.com/nilab/CellTypeSpecificAccessibilityPrediction code is not accessible. Could the authors please share the code as well? I would expect this repository to allow researchers to easily re-evaluate new models on the benchmarks posed by the manuscript.

We apologize that there was a typo in the Github link in our original manuscript. We have fixed this error, and the Github can be accessed here: https://github.com/ni- lab/CellTypeSpecificAccessibilityPrediction.

Minor:

1. L71 "accessibility characterizes active CREs,". Accessible CREs are not necessarily transcriptionally active. Would therefore suggest saying 'potentially active CRE'

We have modified the text to reflect this suggestion.

2. L99: "...provide a comprehensive view of the performance of current state-of-the-art genomic deep learning models in CREs" - The evaluation doesn't consider comparison to say MPRA data which means that it's not comprehensive. The authors should therefore remove the "comprehensive" claim.

We have modified the text to reflect this suggestion.

3. Fig. 2A: The authors should also attempt to add the performance of experimental replicates. Some of the peaks accessible only in a single cell type might be technical artifacts. If experimental replicates are not available, then the authors should use the most similar track as a substitute for the experimental replicate.

We have added a supplementary figure (Fig. S3) showing a comparison to the performance of experimental replicates for five of the Enformer accessibility tracks.

4. Fig. 2A: When quantifying the cell type specificity of predictions, the authors should also look at alternative metrics such as precision per CRE for the top N predictions, where N is the number of accessible cell types for that cell type. Note that one should z-score normalize the predictions for each predicted experiment first before doing this comparison. Another relevant

evaluation metric would be to look at how well the models can predict differentially accessible regions given a specific pair of cell types.

We have added two additional analyses to provide more insight into the cell type specificity of the model predictions. First, we have taken the reviewer’s suggestion to look at the precision per peak for the top N predictions for Enformer (Fig. S1). In general, we find the cell type precision of Enformer’s predictions to be low, although a small fraction of peaks are predicted with high precision, likely corresponding to more ubiquitously accessible peaks. In addition, we have quantified the false negative and false positive rates of the multi-task and single-task models trained on the Loeb, et al. and Calderon, et al. datasets, as suggested by Reviewer #4 (Fig. S14). We find that the multi-task models tend to have higher false positive rates, meaning that they predict peaks to be active in incorrect, or additional, cell types. For both types of models, we also observe that the false negative rate is higher for cell type specific peaks than ubiquitous peaks. We observe a tradeoff between the false positive and false negative rates, with single-task learning generally reducing the false positive rate – consistent with our observation that single-task model predictions are generally less over-correlated between cell types – but slightly increasing the false negative rate, suggesting that some peak predictions may benefit from information sharing across cell types. We believe that these analyses provide additional insight and validation of the poor cell type specific prediction of current genomic deep learning models.

5. Fig. 2B: How much of that figure can be explained by distance from TSS or element types (promoter vs enhancer)?

In Supplementary Figure 5, we have plotted the TSS distance distributions (Fig. S5c) and effect size distributions (Fig. S5d) for the eQTLs in each cell type specificity bin. There is a slight trend towards eQTLs in the most cell type specific bin being more distal, which may be related to some of the drop in performance. This drop cannot be explained by distance between the variant and predicted output, however, as we obtained Enformer and Sei predictions for these eQTLs centered at the variant, not at the TSS. Please also see our response below to Reviewer #4, point 6 for discussion of a similar point.

6. Fig. 2C is introduced before Fig. 2a (L123 vs L129)

Thank you for pointing this out. We have modified the order of figure panels to correctly reflect the order they are introduced in the text.

7. Fig. 2D should also contain the bar showing the enrichment from 2C for easier comparison to all/non-stratified variants using Enformer. Figure legend should also mention 'Low/high Enformer SAD scores'. The authors should also mention that using variants in more cell type specific peaks with high Enformer SAD score are even more highly enriched for trait heritability than variants in cell type specific peaks generally.

We have modified Figure 2D and the accompanying figure legend and text for clarity based on the reviewers’ suggestions. In particular, we have replaced the bar showing enrichment of ‘Low Enformer SAD score’ variants with the bar from Fig. 2C (now Fig. 2A) showing the enrichment of all variants, as we believe this is a more useful comparison which directly shows that the subset of variants with high Enformer SAD scores in cell type specific peaks are more enriched

for trait heritability than all variants in cell type specific peaks. We have also modified the figure legend to explicitly mention “high Enformer SNP Accessibility Difference (SAD) scores.” In addition, we have modified the accompanying results text to read: “For the majority of tested traits, we find that variants in cell type specific peaks with high Enformer SAD scores are more ~~highly~~ enriched for trait heritability **than all variants in cell type specific peaks generally**.“

8. L179: "Taken together, …these results indicate… that models have decreased … accuracy."

-> I don't think Fig. 2D is showing lower performance in cell type specific CREs. Therefore, I would suggest moving the Fig. 2C and D to a separate section with its own conclusion sentence.

We appreciate that this wording was ambiguous and have modified the text to include separate conclusion sentences for the eQTL versus GWAS benchmarks. We agree that the results on the GWAS benchmark in Fig. 2D demonstrate that Enformer’s predictions in cell type specific peaks are at least somewhat informative for the task of identifying complex trait-relevant variants. (It is more difficult to draw conclusions about their informativeness in the less cell type specific peaks due to the low overall enrichment of heritability in these peaks for most of the evaluated traits.) We have clarified in the text that the overall takeaway about variant effect prediction performance in cell type specific peaks is somewhat inconclusive, and may depend on the specific task or phenotype of interest.

9. Fig. S3A y-axis should be shown in log-scale.

We agree that this y-axis of Fig. S3A (now Fig. S5C) should be shown in log-scale. We have updated the figure accordingly.

10. L87: "...we reasoned that negative transfer led to sub-optimal performance": There might be other reasons beyond negative transfer for multi-task models to not be performing as well such as limited capacity. Have the authors carefully re-trained the multi-task vs single-task models while matching for the right model capacity to demonstrate the impact of negative transfer?

We appreciate this point and have addressed it as part of our response to Major point #5 above. We have also removed the specific language about negative transfer on L87.

11. Fig. 3A: Why is the y-axis in different units (proportion heritability) vs Fig. 2C,D (enrichment). We have updated Figure 3A to report enrichment, to be consistent with Figure 2.
12. L212: "All models achieve a genome-wide reference accuracy comparable to previous work (Fig. S6, Fig. S7)". Which of the curves in Fig. S6, S7 is showing previous work?

We apologize for the confusing wording, as we did not mean to suggest that Fig. S6 and Fig. S7 (now Fig. S11 and S12) contained curves showing the performance of previous work. Rather, the genome-wide accuracies of our models (“all sequences”) reported in those figures are comparable to performance measures reported in previous work. We have updated our text to clarify this point.

13. Figures S6, S7 are introduced before Fig. S4 in the text. Also, single-task models haven't been mentioned until then.

We have updated the text to remove the early reference to Figures S6 and S7 and have instead referenced a new supplementary table, Supplementary Table 1 (which also more clearly documents the performance of all of our models on each of the peak sets and benchmarks).

14. L208: Do the authors train only single-task models or multi-task models here?

In the analysis introduced on L208 in the original manuscript, we train only multi-task models. We have added additional text to the introductory sentence of this paragraph (highlighted in bold below) to clarify this: “For each dataset, we then train a set of **multi-task** convolutional neural networks (CNNs) to map input DNA sequences (1344bp) to continuous measures of chromatin accessibility (normalized ATAC-seq read counts) **in each cell type.**”

15. Fig. 3: Is the model compared here multi-task or single-task? I've had trouble figuring this out from the figure legend / text.

The models compared in Figure 3 are multi-task models for each dataset trained on all cell types in that dataset (i.e. three replicate multi-task models for Loeb, et al., three replicate multi- task models for Calderon, et al.). We have clarified this in the figure legend and text.

16. Fig. 3C vs 4A -> consistent colors should be used for multi-task prediction models since they are showing the same performance numbers or?

We use the following color scheme throughout Fig 3C/D and Fig 4, as well as the corresponding supplementary figures:

|  | Multi-task | Single-task |
| --- | --- | --- |
| Cell type specific peaks | Dark blue | Light blue |
| Ubiquitous peaks | Dark orange | Light orange |

17. L222: "...model trained on related cell types also have decreased performance…" - what do the authors mean by 'trained on related cell types'? Weren't the models trained in the same cell type as they are being evaluated on? -> After re-reading the text a few times I realized the text was actually talking about a multi-tasking model. The authors should make that more explicit in the text and include 'multi-task' in the title of this section.

We apologize for the confusion and have added additional text, as well as changed the title of the relevant section, to make it more clear that the evaluations are being performed on multi- task models trained on closely related cell types (i.e. only kidney cell types or only immune cell types). In particular, we have changed the section title from “Poor cell type specific accessibility prediction on related cell types” to “Multi-task models trained on related cell types exhibit poor cell type specific accessibility prediction.”

18. Fig. 3E,F -> The authors should also show the same prediction example for the single-task models discussed in Fig. 4.

We have added the same prediction example for the single-task models as Fig. S18.

19. Fig. S6, S7 are very difficult to read. The authors should consider splitting these out into multiple panels.

We have updated the formatting of Figures S6 and S7 (now Figures S11 and S12) for clarity. In particular, we have taken the reviewer’s suggestion and split out the results for each set of evaluation regions – cell type specific peaks, ubiquitous peaks, and all sequences – into separate figure panels. In addition, we have used different ticks to denote single-task and multi- task models to limit redundancy in the figure legend, as suggested by another reviewer.

# Reviewer #3:

In this manuscript, the authors identified a limitation in current genomic deep learning models: the accuracy of these models decreases in cell type-specific CREs. This includes both prediction accuracy for the reference sequence and for variant effects. The authors then compare single-task models to multi-task models and report an improvement in cell-type- specific CRE predictions. Overall, this is a useful benchmark study that contributes to our understanding of genomic deep learning model performance. However, I have the following concerns:

Major points:

1. The authors observed an over-correlation between model predictions for different cell types compared to experimental measurements. While it is plausible that this over-correlation is partially due to model bias, another potential source of over-correlation is experimental measurement noise. With noisy experimental measurements, the correlation between cell types is expected to be lower than between predictions, even if the predictions are unbiased. Therefore, over-correlation should not be simply considered as a bias of the models. The effect of experimental noise should ideally be taken into account in the analysis.

We agree that measurement noise contributes to under-correlation between experimental measurements and thus to apparent over-correlation between model predictions when compared to experimental measurements. However, measurement noise does not appear to explain the extent of over-correlation that we observe for the model predictions between different cell types. In particular, we use the correlation between experimental measurements from different individuals (for the same cell type) to provide an approximate measure of experimental measurement noise, since both the Loeb, et al. and Calderon, et al. datasets contain data from multiple individuals. Because this cross-individual correlation encompasses both biological and experimental sources of variability, it represents an upper bound on experimental noise. We have added these data as a supplementary figure (Fig. S10). Although a coarse approximation, the gap between the cross-individual correlations and a perfect correlation of 1 can be used to estimate the amount of apparent over-correlation in model predictions that may be due to measurement noise. For example, for Loop of Henle cells, we observe a median cross-individual correlation in cell type specific peaks of approximately 0.65 (Fig. S10), suggesting that a maximum of 0.35 of the gap between measurement and prediction correlation may be attributable to measurement noise. For the multi-task model, the median correlation in measurements between cell types is approximately 0.15 while the median correlation in predictions between cell types is approximately 0.85 (Fig. 4B). Therefore, after taking into account the role of measurement noise, the multi-task model predictions are still considerably over-correlated relative to experimental measurements.

2. The comparison between single-task and multi-task models is informative, but the results are not entirely conclusive in my opinion. The improvement of single-task models in cell type- specific CRE predictions is mild and inconsistent across all cell types. Additionally, the effect on variant effect prediction in cell type-specific CREs is mixed, as shown in Figs. S6b and S7b. The

performance of single-task models on ubiquitous peaks seems to be similar or slightly worse overall. Although the single-task model demonstrates a partial reduction in over-correlation compared to the multi-task model, it is unclear whether this reduction is an improvement or a result of increased variance across single-task models. Moreover, the influence of the number of parameters on the results warrants investigation. It might be worthwhile to explore scenarios where the sum of all single-task model parameters is equal to that of the multi-task model, and where each individual single-task model has the same number of parameters as the multi-task model.

We agree with the reviewer that these are important considerations in fairly evaluating the multi- task versus single-task modeling paradigm. In response to this suggestion and a similar suggestion from Reviewer #2, we have added an experiment to help disentangle the respective roles of increased model capacity versus negative transfer in the performance improvements seen for single-task models (new Fig. S15 and new comparisons in Fig. 4 and Fig. S9). Please see the above response to Reviewer #2, comment 5 for more details. Briefly, these new results provide evidence that the benefit we observe from single-task models may largely be due to increased model capacity, and we have modified the conclusions to more generally recommend increasing model capacity to improve cell type specific performance.

In addition, we agree with the reviewer’s observation that for variant effect prediction in particular, the effect of single-task models is inconsistent. To more accurately reflect these results, especially because variant effect prediction is an important downstream task for these models, we have modified the conclusions to more clearly convey our findings that improvements in cell type specific peak “reference accuracy” do not always translate to improvements in variant effect prediction. We believe that exploring potential reasons for the lack of correlated improvements in these two metrics is an interesting area for future work.

Minor points:

1. The figure panels are not ordered in the same order as they are mentioned in the text. For example, Figure 2c is referenced before Figure 2a in the text.

Thank you for pointing this out; we have corrected the order of figure panels to reflect the order in which they are mentioned in the text.

2. One of the two datasets primarily used in the experiments (Loeb et al.) is not yet publicly available.

The Loeb et al. data have been deposited to GEO, and we will share the link to the data on GEO as soon as it is available (within 5 days of GEO submission).

3. The models trained on Calderon et al. and Loeb et al. are not available.

We have now uploaded the models trained on the Calderon et al. and Loeb et al. data to Zenodo (10.5281/zenodo.10729956).

# Reviewer #4:

Review: Current genomic deep learning models display decreased performance in cell type specific cis regulatory elements

The manuscript's premise is that DNA sequence-based multi-task deep learning (DL) predictors generally focus on genome-wide accuracy. However since disease heritability is enriched in CREs, it is important to assess whether these models are equally accurate in CREs with varying degrees of cell-type specificity. Here, the authors assess the performance of two multitask models (Sei and Enformer) at predicting cell-type specific accessibility and conclude that both models display decreased performance over cell-type specific CREs when compared to shared

/ ubiquitous CREs.

This is an interesting perspective and I believe that this an important assessment to perform as we begin utilizing sequence-based DL models to evaluate genetic variation. However, in its current form, the evidence provided in order to reach the stated conclusions is insufficient and a more complete analysis would make this research more useful.

Concerns:

1. The first major claim in the paper is that cell-type specific accessibility peaks are more enriched for heritability when compared to shared CREs ( Fig. 2C, D). This conclusion is based on stratified LD score regression (S-LDSC) which partitions heritability given a set of annotations. The authors perform S-LDSC by including the baseline LD annotation set. The baseline LD annotations contain several annotations that would overlap extensively with shared CRE features but not cell-type specific CREs - such as FANTOM 5 enhancers, promoters etc. Since the heritability estimates provided by S-LDSC are conditional estimates, it is unclear whether the decreased heritability in shared CREs is an artifact driven by the inclusion of other annotations marking these regions. Performing S-LDSC without conditioning on noncoding baseline annotations would provide an unbiased estimate of heritability enrichment in shared vs. cell-type specific CREs.

We appreciate this point and have performed a comparison of S-LDSC conditioned on the full set of baseline annotations (as originally reported in the paper) to S-LDSC after removing all baseline annotations corresponding to promoter and enhancer marks from the conditioning. When not conditioned on promoter/enhancer baseline annotations, the relative enrichment of heritability in accessibility peaks with high versus low cell type specificity decreases slightly (see figures below), illustrating the reviewer’s point that conditioning on these baseline annotations biases the enrichment comparison between the two peak sets. However, even when not conditioning on promoter/enhancer baseline annotations, we observe that peaks with high cell type specificity are more enriched for trait heritability than peaks with low cell type specificity.

We have updated the relevant figures in the manuscript to show enrichment results that are not conditioned on promoter/enhancer baseline annotations, to provide a more unbiased estimate of the relative heritability enrichment in shared vs. cell type specific peaks.

S-LDSC conditioned on all baseline annotations:

S-LDSC not conditioned on promoter/enhancer baseline annotations:

2. In Fig. 2D, loci are divided into "high" vs. "low" magnitude Enformer variant scores and heritability enrichment is computed for each category. How is a threshold chosen to divide predictions into categories? Computing statistical significance & computing heritability enrichment for significant vs. non-significant variant effects would be more convincing than using a single threshold to categorize variant effects.

In the main figure, we use the median Enformer variant score as the threshold to divide variants into “high” vs. “low” scores, as more stringent thresholds reduce the number of variants and the size of the annotation set for S-LDSC, increasing the standard error of the enrichment estimates. To address the reviewer’s concerns, we vary our choice of threshold and find that the increase in heritability enrichment observed for “high” Enformer SAD score variants is largely consistent when using more stringent thresholds. (Note that we do not compute the statistical significance of a given score threshold because Enformer’s variant scores don’t contain any uncertainty or confidence estimates.) In Fig. S6 of the revised manuscript, we show a replicate of our enrichment analysis using the top 10% of Enformer SAD scores (compared to the top 50% used in Fig. 2D). Note that with this choice of threshold, the standard error of the S-LDSC enrichment estimates becomes quite large, so we still use the 50% threshold in the main figure. Also note that in response to a comment from Reviewer #2, we have replaced the bar showing enrichment of ‘Low Enformer SAD score’ variants with the bar from Fig. 2A (previously Fig. 2C) showing the enrichment of all variants, as we believe this is a more useful comparison which directly shows that the subset of variants with high Enformer SAD scores in cell type specific peaks are more enriched for trait heritability than all variants in cell type specific peaks.

3. In Fig. 3A, the author's measure the proportion of heritability within cell-type specific vs. shared CREs for the two ATAC-seq datasets. However, the proportion of heritability will be higher for a category if it contains a greater proportion of SNPs. It would be nice to see

heritability enrichment reported here (controlling for number of SNPs in annotation). This would also be consistent with Fig. 2C, which already reports heritability enrichment.

We have updated Fig. 3A to report heritability enrichment instead of proportion of heritability.

4. In Fig. 3C, accuracies over cell-type specific CREs are reported for 4 and 3 cell-types from the Loeb at al. and Calderon et al. datasets shown. For a more complete evaluation, it would be nice to see the results for all cell-types, this could be added to supplementary figures.

We have added the results for all cell types in Supplementary Table 1.

5. Figure 3D: While assessing prediction uncertainty, is the coefficient of variation (uncertainty in prediction) significantly different between cell-type specific vs. shared CREs, especially in the Calderon et al. set?

We find that the coefficient of variation is significantly different between cell type specific and shared peaks for both datasets (under a one-sided Mann-Whitney U test, FDR < 0.05). We have updated the figure to display statistical significance.

6. The regulatory grammar at TSSs is distinct from that at distal enhancers - is the model's ability to learn cell-type specificity dependent on whether the CRE is a proximal element such as a TSS or distal enhancer?

We agree with this important point and have added a supplementary figure (Fig. S4) in which we stratified peaks into three equally sized TSS distance bins and looked at performance across all peaks, as well as within each cell type specificity bin. We find that across all peaks, there is a slight decrease in performance for more distal peaks compared to more proximal peaks (Fig.

S4a). Importantly, however, the trend of decreased performance in more cell type specific peaks seems to be independent of TSS distance, as model predictions in the most cell type specific peaks are similarly poor regardless of whether the peaks are proximal or distal (Fig. S4a). We also show the proportion of sequences in each cell type specificity bin that are in each TSS distance bin (Fig. S4b). Unsurprisingly, the most cell type specific peaks are more skewed towards distal sequences, which explains some of the difference in overall performance at different TSS distances.

7. For specific peaks which are mis-predicted, are false negatives or false positives the problem? If these peaks are false positives, what motifs are driving the predictions? To assess this, analyzing model attributions would be useful.

We appreciate this suggestion and have added an analysis of the false negative and false positive rate of predictions, as well as an analysis of model attributions for specific peaks which are mis-predicted. We find that the multi-task models trained on the Loeb, et al. and Calderon, et al. datasets tend to have higher false positive rates, meaning that they predict peaks to be active in incorrect, or additional, cell types (Fig. S14). For both multi-task and single-task

models, we also observe that the false negative rate is higher for cell type specific peaks than ubiquitous peaks. We observe a tradeoff between the false positive and false negative rates, with single-task learning generally reducing the false positive rate – consistent with our observation that single-task model predictions are generally less over-correlated between cell types – but slightly increasing the false negative rate, suggesting that some peak predictions may benefit from information sharing across cell types.

We also performed an attribution analysis for specific peaks with false positive predictions to examine the motifs driving the predictions. In the Loeb, et al. data, we highlight a Stromal cell specific peak that is mispredicted by the multitask model to be a peak in the other cell types as well, but is correctly predicted by the single-task models to only be accessible in Stromal cells. In silico mutagenesis of this peak reveals that a TEAD-like motif is driving the prediction of the multi-task model in Stromal and non-Stromal cell types (Fig. S17a). By contrast, this TEAD-like motif is less apparent in the single-task models trained on the non-Stromal cell types. In the Calderon, et al. data, we highlight a dendritic cell specific peak that is mispredicted by the multitask model to be a peak in the other cell types as well, but is correctly predicted by the single-task models to only be accessible in dendritic cells. In silico mutagenesis of this peak reveals that a SPIB-like motif is weakly driving the predictions of the multi-task model in dendritic and non-dendritic cell types (Fig. S17b). By contrast, this SPIB-like motif is only driving predictions of the single-task dendritic cell model.

8. Why is cell type specific performance worse? The authors seem to favor model bias, but several alternatives are worth considering:

8a. Ubiquitous peaks have a greater true positive rate because specific peaks are more likely false positive peak calls or attributable to genetic variation outside the reference. Authors could assess using ENCODE replicates whether ubiquitous peaks are more likely to appear in multiple replicates.

We thank the reviewer for this suggestion. For five of the Enformer accessibility tracks with ENCODE replicates, we have added a comparison of Enformer’s performance to the correlation between replicates within different cell type specificity bins (Fig. S3). We find that although cell type specific peaks are slightly less replicable than ubiquitous peaks, the difference does not explain the drop in performance in these peaks.

8b. Do ubiquitous peaks have greater information content than specific peaks? I.e. CpG island promoters and CTCF insulators are very easy to predict. Are specific peaks more likely to be driven by complex grammars that are inherently more challenging for ML models to learn?

We agree with the reviewer that certain sequence features, motifs, or sequence grammars may make ubiquitous peaks easier to predict than cell type specific peaks, and we appreciate the suggestions for testing such features. We have now looked specifically at both GC content, CpG islands and CTCF motifs, as suggested by the reviewer, as well as performed an unbiased motif enrichment analysis to identify TF motifs enriched in cell type specific versus ubiquitous peaks. We have added these results to the manuscript in Fig. S8. In both the Loeb et al. and Calderon

et al. datasets, we find that ubiquitous peaks have slightly higher GC content than non- ubiquitous peaks (Fig. S8a) and that a substantially larger proportion of ubiquitous peaks contain putative CpG islands (Fig. S8b) and CTCF motifs (Fig. S8c). Our motif enrichment analysis identifies several TFs previously known to be active in the studied cell types (Fig. S8d); for example, we identify HNF4A as enriched in proximal tubule peaks, SPI1 as enriched in myeloid cell peaks, and Jun as enriched in T cell peaks. Interestingly, in the Loeb et al. data in particular, we find stronger enrichment of motifs in ubiquitous peaks than cell type specific peaks (Fig. S8d), which supports the hypothesis that there may be more information content in these ubiquitous peaks, or that they have a higher signal to noise ratio. We have added a discussion of these points to the revised manuscript.

Additional Minor Concerns:

Figure 1A & 1B: Number of cell-types reported on the x-axis is incorrect. There are 684 DNase tracks in the Enformer/ but not all of them are distinct cell-types. For example, there might be several K562 tracks from distinct sources. Similarly, the legend is also incorrect in S1A and S1B.

We thank the reviewer for pointing this out. We were indeed referring to the number of DNase tracks, and the 684 DNase tracks contain several cases where there are multiple tracks for a particular cell type, so the x-axis label “Number of cell types” was incorrect. We have modified the x-axis label in the relevant figure panels to read “Number of chromatin accessibility profiles with a peak” to more accurately reflect the information being visualized.

**Second round of review**

**Reviewer 1**

1. The data deposited in GEO should be made available not only with the publication of the manuscript, but also for the review process (could be made available only for the reviewers). In addition, the GitHub repository code lacks the modifications that resulted from responses to the review comments, e.g. a multi-task model with increased capacity.
2. We have concerns regarding the interpretability techniques applied in the revised manuscript.
a. Global interpretability – we were confused by the description of this interpretability technique. The authors wrote that they implanted the motif in background and dishuffeled background sequences. In this first case, the sequence content is of background – why didn’t the authors use the active regions rather than background? And in the second, dishuffeled sequences are synthetic sequences and thus do no represent true biology. We recommend more established techniques, such as TF-MoDISco (Shirkumar et al. Arxiv 2018), to discover re-occurring regulatory elements in attribution maps.
b. Local interpretability – the differences may only be in the scale (as the plots share the same y-axis limits) and not in the trends, thus not really showing a difference between multi-task and single-task models. Moreover, we recommend using other local interpretability techniques that have been highly successful in genomics, such as DeepLift (Shirkumar et al. Arxiv 2017), Enhanced Integrated Gradients (Jha et al. Genome Biology 2020), and scramblers (Linder et al. Nature Machine Intelligence 2022), instead of in silico mutagenesis, which has its limitation compared to the gradient-based approaches.
c. Model capacity – as the authors have shown in the revision that multi-task models with increased capacity are as good as single-task models in specific CREs, they should also use these increased capacity models in the interpretability evaluation.

**Reviewer 2**

The authors have addressed all my major points - great job. I have a few remaining follow-up points I would ask the authors to address:

1. Re Point 10:
> We appreciate this point and have addressed it as part of our response to Major point #5 above. We have also removed the specific language about negative transfer on L87.

In the introduction, the authors still say refer to negative transfer:
"We reasoned that negative transfer between cell types could contribute to poor performance on cell type specific epigenetic marks, including cell type specific accessible regions. Therefore, using custom models trained and evaluated on cell type specific ATAC-seq data from primary kidney and immune cells, we also evaluate the effect of single-task versus multi-task learning on predictive accuracy in cell type specific accessible regions."

I would suggest the authors remove the mention of negative transfer in the introduction and potentially move it to the discussion or the results section when discussing what might be causing lower performance. This speculation in the introduction is misleading since it's not really followed up or demonstrated in the paper.

2. In Figure 2: the x axis is of panel A is divided into High/Low cell type specificity. However, figure B is stratified by the number of cell types with a peak. Is it possible to use the exact same set to evaluate the performance of the model in Figure 2B and S2 as well?

3. Figure S19 should be shown as Figure 4C. It contains one extra bar plot in the figure and puts the single task training process into perspective of alternative methods.

**Reviewer 3**

The authors have addressed my previous concerns. I thank the authors for the revision.

**Reviewer 4**

The revisions have addressed a majority of my concerns. I believe the manuscript provides valuable insights into the behavior of multitask models at cell-type specific loci.

Minor points to address:

1. Include a definition of negative transfer. Negative transfer is referred to using a couple of citations in the introduction but no definition is provided.

2. It is not clear that Supp. Fig. 17A supports the claim it is used to make. The argument is that a TEAD-like motif is driving predictions for in both stromal and non-stromal cell types for multitask models, but only in stromal cell-types for the single-task models. However, from Supp. Fig. 17A, it looks like TEAD is not picked out by the single-task model at all, attributions at the TEAD motif are similar to or lesser than it's the surrounding nucleotides. Is it likely that the region looks similar in terms of attribution score distributions across cell-types and only differs in scale. I'd recommend providing a different example or using TF-MoDISco to do a global analysis in order to support this claim, or removing this claim.

3. Add in values for the y-axis in Fig. 3e, f.

4. The work relies on the Loeb et al. dataset, but this work is unpublished. Provide a preprint / GEO accession.

**Authors’ response to reviewers**

We thank the reviewers for their additional comments. Based on this feedback, we have carried out an additional interpretability analysis of the single-task and multi-task models using TF- MoDISco, which we have added to the manuscript in Fig. S16C,D. We have also addressed a number of more minor points and clarifications suggested by the reviewers below.

|

# Reviewer #1:

1. The data deposited in GEO should be made available not only with the publication of the manuscript, but also for the review process (could be made available only for the reviewers). In addition, the GitHub repository code lacks the modifications that resulted from responses to the review comments, e.g. a multi-task model with increased capacity.

We have now updated the manuscript with the GEO accession number for the Loeb et al. data and have updated the GitHub repository to include all code used for analyses added during the revision.

2. We have concerns regarding the interpretability techniques applied in the revised manuscript.
   a. Global interpretability – we were confused by the description of this interpretability technique. The authors wrote that they implanted the motif in background and dishuffeled background sequences. In this first case, the sequence content is of background – why didn’t the authors use the active regions rather than background? And in the second, dishuffeled sequences are synthetic sequences and thus do no represent true biology. We recommend more established techniques, such as TF-MoDISco (Shirkumar et al. Arxiv 2018), to discover re- occurring regulatory elements in attribution maps.

We apologize for the lack of clarity in the description of the motif insertion analysis. We used dinucleotide shuffled peak sequences **as** the background sequences that motifs were inserted into. To provide more clarity, we have graphically depicted the procedure we followed below:

Peak sequence	Shuffled sequence	Motif-inserted

sequence

**Motif activity score:****-**

Motif-inserted

sequence

Shuffled sequence

To address the second point, we have performed an additional analysis using TF-MoDISco to identify motifs driving predictions of the single-task and multi-task models in cell type specific and ubiquitous peaks, which we have added to the manuscript in Fig. S16C,D. For the baseline multi-task, high capacity multi-task, and single-task models, we compute the Jaccard similarity in the motifs identified by TF-MoDISco across cell types. We find that the motifs driving single- task and high capacity multi-task model predictions in cell type specific peaks tend to be more distinct across cell types, supporting the conclusions from the motif insertion analysis that some of the over-correlation between cell types that we observe for the baseline multi-task models may be due to the models learning a similar regulatory syntax for distinct cell types.

b. Local interpretability – the differences may only be in the scale (as the plots share the same y-axis limits) and not in the trends, thus not really showing a difference between multi-task and single-task models. Moreover, we recommend using other local interpretability techniques that have been highly successful in genomics, such as DeepLift (Shirkumar et al. Arxiv 2017), Enhanced Integrated Gradients (Jha et al. Genome Biology 2020), and scramblers (Linder et al. Nature Machine Intelligence 2022), instead of in silico mutagenesis, which has its limitation compared to the gradient-based approaches.

We thank the reviewer for this feedback and have added additional text to the results section to make it clear that some of the differences between multi-task and single-task *in silico* mutagenesis (ISM) scores may be due to scale. We plot ISM scores on the same scale for all models, since we generally observe that the models make predictions on similar scales for held- out reference sequences.

We also appreciate the reviewer’s suggestion of additional attribution methods that are popular in genomics. Based on observations in previous publications (Zheng, et al. *Nature Machine Intelligence*, 2021; Cochran, et al. *Genome Research*, 2022) and a recent review paper (Novakovsky, et al. *Nature Reviews Genetics*, 2023), we have chosen to use ISM for our local interpretability analysis because the benefits of other attribution methods seem to largely be computational efficiency, which was not a bottleneck in this case.

c. Model capacity – as the authors have shown in the revision that multi-task models with increased capacity are as good as single-task models in specific CREs, they should also use these increased capacity models in the interpretability evaluation.

We have added results from the increased capacity models to the interpretability evaluation (Fig. S16 and S17) and example predictions (Fig. S18).

# Reviewer #2:

The authors have addressed all my major points - great job. I have a few remaining follow-up points I would ask the authors to address:

1. Re Point 10:

> We appreciate this point and have addressed it as part of our response to Major point #5 above. We have also removed the specific language about negative transfer on L87.

In the introduction, the authors still say refer to negative transfer:

"We reasoned that negative transfer between cell types could contribute to poor performance on cell type specific epigenetic marks, including cell type specific accessible regions. Therefore, using custom models trained and evaluated on cell type specific ATAC-seq data from primary kidney and immune cells, we also evaluate the effect of single-task versus multi-task learning on predictive accuracy in cell type specific accessible regions."

I would suggest the authors remove the mention of negative transfer in the introduction and potentially move it to the discussion or the results section when discussing what might be causing lower performance. This speculation in the introduction is misleading since it's not really followed up or demonstrated in the paper.

We thank the reviewer for this insight and have altered this section of the introduction to address their concern. In particular, we have more broadly introduced how both negative transfer and limitations in model capacity could impact the performance of multi-task models, and how our evaluation strategy can evaluate the impact of both of these phenomena.

2. In Figure 2: the x axis is of panel A is divided into High/Low cell type specificity. However, figure B is stratified by the number of cell types with a peak. Is it possible to use the exact same set to evaluate the performance of the model in Figure 2B and S2 as well?

Below, we show the results of redoing the analysis in Figure 2B using the exact same data splits used in Figure 2A. These results support the conclusions drawn from Figure 2B, that Enformer performance is worse on high cell type specificity peaks compared to low cell type specificity peaks. We have chosen not to add this additional figure to the paper as we believe it conveys similar information to what is already shown in Figure 2.

3. Figure S19 should be shown as Figure 4C. It contains one extra bar plot in the figure and puts the single task training process into perspective of alternative methods.

We appreciate the reviewer’s perspective. The main point conveyed in Figure 4 is how different methods for training on the same dataset (i.e. single-task versus multi-task learning) affect performance in cell type specific peaks. The comparison to Enformer, which has a much larger architecture and trains on many more datasets, makes a separate and unrelated point. Since we believe the Enformer comparison does not meaningfully contribute to the message of Figure 4, we have left it in Fig. S19 and clarified the discussion of this comparison in the main text.

# Reviewer #3:

The authors have addressed my previous concerns. I thank the authors for the revision.

# Reviewer #4:

The revisions have addressed a majority of my concerns. I believe the manuscript provides valuable insights into the behavior of multitask models at cell-type specific loci.

Minor points to address:

1. Include a definition of negative transfer. Negative transfer is referred to using a couple of citations in the introduction but no definition is provided.

We appreciate this suggestion and have added a brief description of negative transfer to the introduction.

2. It is not clear that Supp. Fig. 17A supports the claim it is used to make. The argument is that a TEAD-like motif is driving predictions for in both stromal and non-stromal cell types for multitask models, but only in stromal cell-types for the single-task models. However, from Supp. Fig. 17A, it looks like TEAD is not picked out by the single-task model at all, attributions at the TEAD motif are similar to or lesser than it's the surrounding nucleotides. Is it likely that the region looks similar in terms of attribution score distributions across cell-types and only differs in scale. I'd recommend providing a different example or using TF-MoDISco to do a global analysis in order to support this claim, or removing this claim.

We thank the reviewer for their feedback and have toned down the language around this point.

3. Add in values for the y-axis in Fig. 3e, f.

We have updated the figure to include y-axis values.

4. The work relies on the Loeb et al. dataset, but this work is unpublished. Provide a preprint / GEO accession.

We have updated the manuscript with a citation to the preprint (https[://w](http://www.biorxiv.org/content/10.1101/2024.06.18.599625v1))ww[.b](http://www.biorxiv.org/content/10.1101/2024.06.18.599625v1))i[orxiv.org/content/10.1101/2024.06.18.599625v1)](http://www.biorxiv.org/content/10.1101/2024.06.18.599625v1)) and the GEO accession number for the Loeb et al. data.
